# miR-309a is a regulator of ovarian development in the oriental fruit fly *Bactrocera dorsalis*

**DOI:** 10.1371/journal.pgen.1010411

**Published:** 2022-09-16

**Authors:** Qiang Zhang, Wei Dou, Clauvis Nji Tizi Taning, Shan-Shan Yu, Guo-Rui Yuan, Feng Shang, Guy Smagghe, Jin-Jun Wang

**Affiliations:** 1 Key Laboratory of Entomology and Pest Control Engineering, College of Plant Protection, Southwest University, Chongqing, China; 2 Academy of Agricultural Sciences, Southwest University, Chongqing, China; 3 International China-Belgium Joint Laboratory on Sustainable Crop Pest Control between Southwest University in China and Ghent University in Belgium, Chongqing, China; 4 Laboratory of Agrozoology, Department of Plants and Crops, Ghent University, Ghent, Belgium; Universidad de Valparaiso, CHILE

## Abstract

Fecundity is arguably one of the most important life history traits, as it is closely tied to fitness. Most arthropods are recognized for their extreme reproductive capacity. For example, a single female of the oriental fruit fly *Bactrocera dorsalis*, a highly invasive species that is one of the most destructive agricultural pests worldwide, can lay more than 3000 eggs during its life span. The ovary is crucial for insect reproduction and its development requires further investigation at the molecular level. We report here that miR-309a is a regulator of ovarian development in *B*. *dorsalis*. Our bioinformatics and molecular studies have revealed that miR-309a binds the transcription factor *pannier* (*GATA-binding factor A*/*pnr*), and this activates yolk *vitellogenin 2* (*Vg 2*) and *vitellogenin receptor* (*VgR*) advancing ovarian development. We further show that miR-309a is under the control of juvenile hormone (JH) and independent from 20-hydroxyecdysone. Thus, we identified a JH-controlled miR-309a/*pnr* axis that regulates *Vg2* and *VgR* to control the ovarian development. This study has further enhanced our understanding of molecular mechanisms governing ovarian development and insect reproduction. It provides a background for identifying targets for controlling important Dipteran pests.

## Author summary

The ovary is a very critical organ for insect reproduction. Especially, many insect pests are famous for their large reproductive capacity. Therefore, understanding the molecular mechanisms involved in ovarian development could significantly contribute in the development of new insect pest control strategies. In this study, we report that miR-309a regulates the development of the ovary in an important dipteran pest, *B*. *dorsalis*, through a transcriptional factor, *pannier* (*GATA-binding factor A*/*pnr*), which in turn directly mediates the expression of yolk *vitellogenin 2* (*Vg 2*) and *vitellogenin receptor* (*VgR*). Moreover, miR-309a is under the upstream control of juvenile hormone (JH). Here, in Dipterans, a novel JH-miR-309a-*pnr*-*Vg*-related genes regulatory pathway was found in ovarian development. This finding advances our understanding of a mechanism regulating insect ovarian development and provides new insights for potential targets to control dipteran pests through the reproductive strategy.

## Introduction

Normal ovarian development is crucial for reproduction. Therefore, uncovering the underlying molecular mechanisms in this process, especially for pest insects with high fecundity, could significantly contribute to devising innovative pest control strategies [1,2]. Ovarian development in insects is primarily controlled by two hormones, juvenile hormone (JH) and 20-hydroxyecdysone (20E). To perform their regulatory roles, JH and 20E, functioning through their corresponding receptors, methoprene-tolerant (Met) and ecdysone receptor (EcR), assert their effects on key genes in ovarian development, *vitellogenin* (*Vg*) and *vitellogenin receptor* (*VgR*) [2]. Vitellogenin(s) provide nutrients for oocytes maturation and embryo development, while VgR is critical for yolk protein internalization into developing oocytes during vitellogenesis [3]. However, the regulatory mechanism of non-coding RNAs in the process of ovarian development still remains unclear.

MicroRNAs (miRNAs) are small, single-stranded non-coding RNA molecules containing approximately 22 nucleotides (nt). They are evolutionarily conserved and play important roles in regulating cellular events at the post-transcriptional level during biological processes such as development, metamorphosis, immunity, reproduction and insecticide resistance [3–5]. Thus, miRNAs have potential to become new prospective targets for insect pest control. The involvement of miRNAs in insect ovarian development have been reported in *Drosophila melanogaster* [6], *Aedes aegypti* [7–9], *Bombyx mori* [3], *Locusta migratoria* [10,11] and *Nilaparvata lugens* [12], and these miRNAs control this process through targets involved in different regulatory pathways (e.g. 20E and JH hormone pathways). However, knowledge on the role of miRNAs in insect ovarian development is still in its infancy and warrants further research.

*Pannier* (*GATA-binding factor A*, also known as *pnr*) is a member of the GATA transcription factor family. Members of the GATA transcription factor family contain one or two zinc finger domains with the amino acid sequence CysX2CysX17CysX2Cys that bind to the DNA sequence (A/T) GATA (A/G) in GATA target genes. GATA factors are evolutionary conserved in mammals, insects, plants, and fungi and involved in cell proliferation, tissue differentiation, immunity and the development of ovaries and embryos [13,14].

The oriental fruit fly, *Bactrocera dorsalis* (Hendel) (Diptera: Tephritidae), is a highly invasive species and is one of the most destructive agricultural pests in the world. It is now prevalent in at least 65 countries [15]. It causes severe economic losses damaging more than 600 kinds of fruits and vegetables. After initial introduction, it disperses widely due to its high reproductive and biotic potentials with up to 10 generations of offspring per year. Under optimal conditions, a female can lay more than 3,000 eggs during its lifetime. Therefore, uncovering the molecular mechanisms behind this high reproductive potential is vital to the development of population control strategies for this pest. Based on the analysis of two transcriptome datasets in our preliminary studies [16,17], miR-309a, a member of a fast-evolving miR-309 cluster [18], appears to be involved in the reproductive process in *B*. *dorsalis*, as it was found to be significantly expressed during ovarian development and was present in the eggs.

Here, we report that miR-309a is a key regulator of ovarian development in *B*. *dorsalis*, and further show that it is involved in a JH-controlled miR-309a/*pnr* axis that regulates *Vg*-related genes in this process. This study provides mechanistic insights into insect ovarian development, and identifies potential targets for pest control aimed at impairing female reproductive capacity.

## Results

### miR-309a is involved in ovarian development in *B*. *dorsalis*

**Fig 1:**
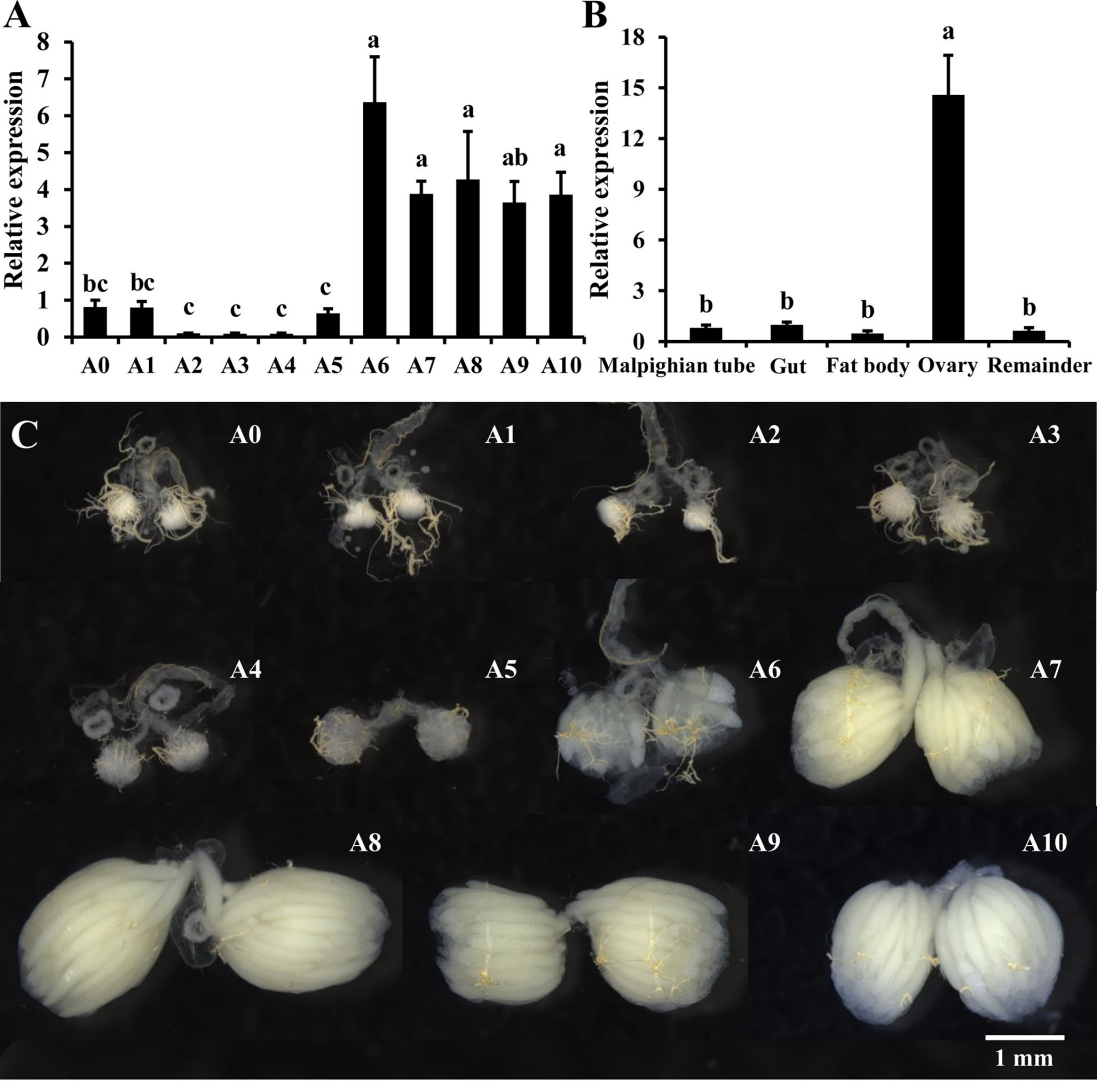
Spatiotemporal expression of miR-309a in *B*. *dorsalis*. (A) Temporal expression of miR-309a in the ovary. (B) Tissue expression of miR-309a, including Malpighian tubules, gut, fat body, ovary, and remainder (the remaining tissue). (C) Ovarian development during A0–A10. A0–A10: ovarian samples of adults from 0 d to 10 d. Data are means ± SE (error bars) of four biological replications. U6 as a reference gene was used to normalize the expression of miR-309a via qBASE software. Different letters above the bars indicate significant differences of miR-309a in different tissues or developmental stages of the ovary (Tukey HSD, ANOVA, *P* < 0.05).

We first examined the temporal expression profile of miR-309a in the ovaries of adult *B*. *dorsalis* from the 1^st^ to the 10^th^ day after eclosion. Our results indicated that the expression of miR-309a is stable from the 1^st^ to the 5^th^ day post-eclosion, after which it increases significantly from the 6^th^ day and remaining at this level till the 10^th^ day post-eclosion (Fig 1A). Then, the tissue distribution of miR-309a in six-day-old female adults was evaluated. The expression level of miR-309a was significantly higher in ovaries than other tissues (Fig 1B), and its expression correlated with ovarian development (Fig 1C). Moreover, *in situ* analyses of miR-309a by miRNA fluorescence *in situ* hybridization (FISH) showed that it highly expressed in follicle cells (S1 Fig).

**Fig 2:**
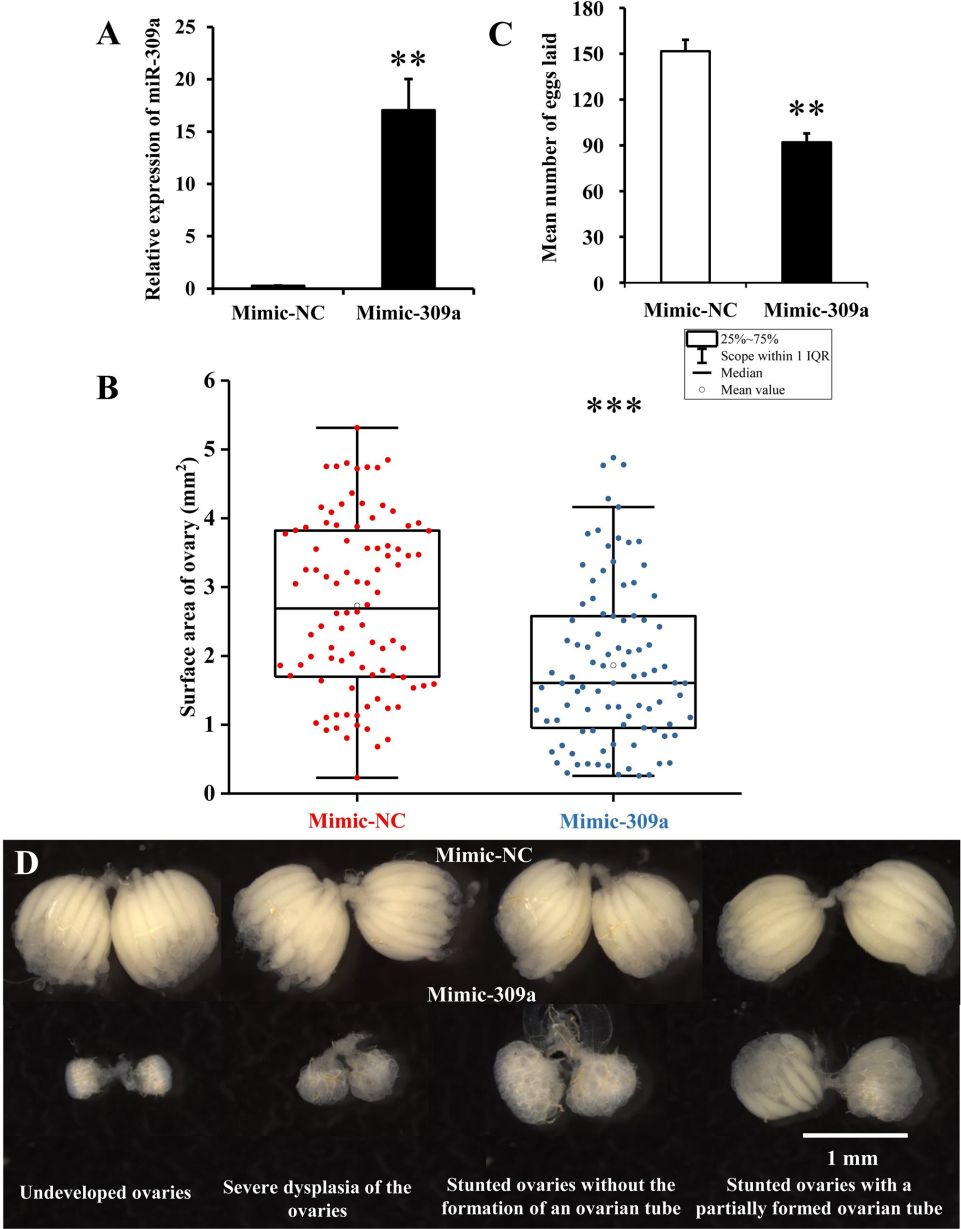
Effect of overexpression of miR-309a on ovarian development in *B*. *dorsalis*. (A) Relative expression of miR-309a after the mimic injection. Data are means ± SE (error bars) of four biological replications. U6 as a reference gene was used to normalize the expression of miR-309a. (B) Surface area of ovary, including mimic-NC (n = 92) and mimic-309a (n = 93) treatments. Data are means ± SE. (C) Egg number. The fecundity of females in the mimic-309a treatment after successfully mating with untreated, naive males. Data are means ± SE of three biological replications. (D) Ovary phenotypes, including undeveloped ovaries, severe dysplasia of the ovaries, stunted ovaries without the formation of an ovarian tube, and stunted ovaries with a partially formed ovarian tube. The differences between means were analyzed by Student’s *t* test. For the significance test: *P < 0.05, **P < 0.01, ***P < 0.001.

We then investigated the effects of the overexpression and repression of miR-309a on ovarian development and oviposition. To this end, three-day-old and four-day-old female adults were injected with 50 pmol of miR-309a mimic (mimic-309a) or antagomir (antago-309a). At 24 and 48 h post-injection, we observed a 67- and 62-fold (*P* = 0.005 and 0.002, respectively) increase in the expression level of miR-309a in the mimic-309a-injected group compared to the mimic-negative control (mimic-NC) (Figs 2A and S2A); a significant reduction with 50% (*P* = 0.039) in miR-309a expression level was observed in the antago-309a-injected group at 24 h post-injection compared to the antago-negative control (antago-NC), but no significant difference was observed at 48 h post-injection (S2A Fig). Hence, we only observed that the phenotypic manifestations in miR-309a-overexpressed female flies showed multiple defects in ovarian development and embryogenesis. At fourth day post-injection with mimic-309a, the surface area of the ovary was significantly (*P* = 2 × 10^−6^) reduced (1.86 mm^2^) compared to the mimic-NC (2.73 mm^2^). Ovarian growth was disrupted after overexpression of miR-309a as manifested by undeveloped ovaries, severe dysplasia of the ovaries, stunted ovaries without the formation of an ovarian tube, and stunted ovaries with a partially formed ovarian tube (Fig 2B, 2C and 2D). Moreover, the average number of eggs laid by the mimic-309a-injected females (92 eggs/adult) over a three-day period was lower (*P* = 0.003) compared to the control (152 eggs/adult) (Fig 2C). These results suggest that miR-309a plays an important role in the ovarian development of *B*. *dorsalis*.

### *pnr* and *facilitated trehalose transporter Tret1 (tret)* as candidate targets of miR-309a

**Fig 3:**
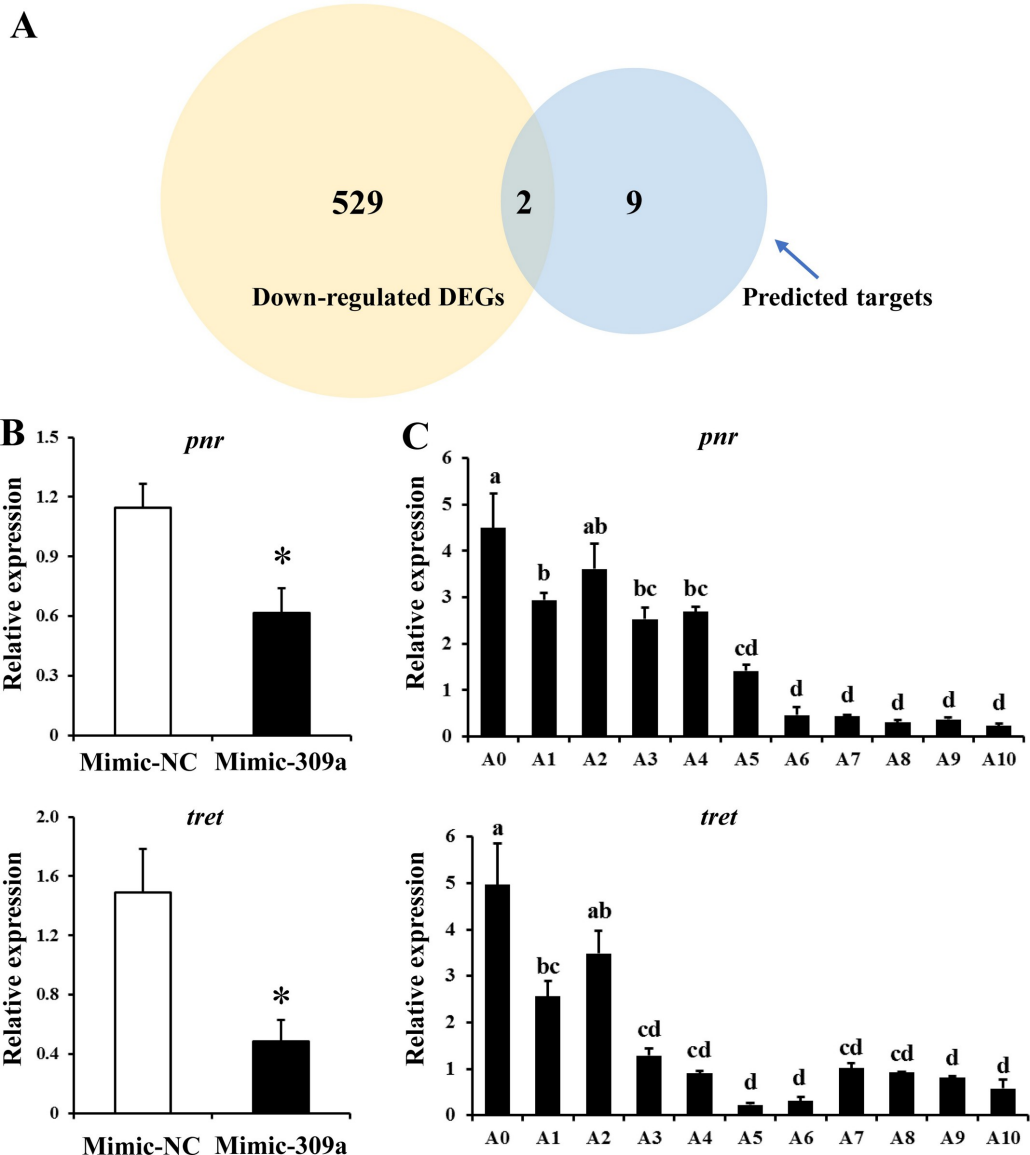
Exploration of miR-309a targets in *B*. *dorsalis*. (A) Venn diagram showing the overlapping down-regulated DEGs after miR-309 overexpression with in silico-predicted target genes by co-analysis using four prediction software programs. (B) Relative expression of the overlapping genes *pnr* and *tret* after miR-309a overexpression. (C) Temporal expression of *pnr* and *tret*. Data are means ± SE (error bars) of four biological replications. *a*-*tubulin* and *rps3* as reference genes were used to normalize the expression of predicted mRNAs. The differences between means were analyzed by Student’s *t* test. For the significance test: *P < 0.05. Different letters above the bars indicate significant differences of *pnr* and *tret* in different developmental stages of the ovary (Tukey HSD, ANOVA, *P* < 0.05).

To investigate the regulatory mechanism through which miR-309a affects ovarian development, we predicted and analyzed potential target genes that could be regulated by miR-309a. A transcriptome analysis of miR-309a-overexpressed female adults was performed, and 836 differentially expressed genes (DEGs) were obtained, with 305 up-regulated and 531 down-regulated DEGs (S1 Data). Then, four different miRNA-target prediction programs were used to identify out 11 putative target genes that could be regulated by miR-309a (S3 Fig and S2 Data). Of these 11 candidate genes, only *pnr* and *tret* were found to be down-regulated DEGs (Fig 3A). Furthermore, the expression levels of *pnr* and *tret* were down-regulated by 46% (*P* = 0.015) and 67% (*P* = 0.013) in ovaries of *B*. *dorsalis* females following the treatment with mimic-309a, respectively, compared to the control (Figs 3B and S4). Additionally, *pnr* and *tret* expression levels were up-regulated by 29% (*P* = 0.030) and 39% (*P* = 0.045) in ovaries of *B*. *dorsalis* females following treatment with antago-309a, respectively, compared to the antago-negative control (S2B Fig). Further quantitative real-time PCR (qPCR) analysis revealed that the temporal expression profiles of these candidate targets were negatively correlated with that of miR-309a in the ovary (Fig 3C). In contrast to the temporal expression profile of miR-309a in the ovary, where the expression of this miRNA spiked later during ovarian development, the expression levels of *pnr* and *tret* were relatively high during early ovarian development and declined as the ovaries matured. These results suggest that *pnr* and *tret* could be potential targets through which miR-309a regulates ovarian development in *B*. *dorsalis*.

### Regulation of *pnr* and *tret* by miR-309a in *in vitro* and *in vivo* assays

**Fig 4:**
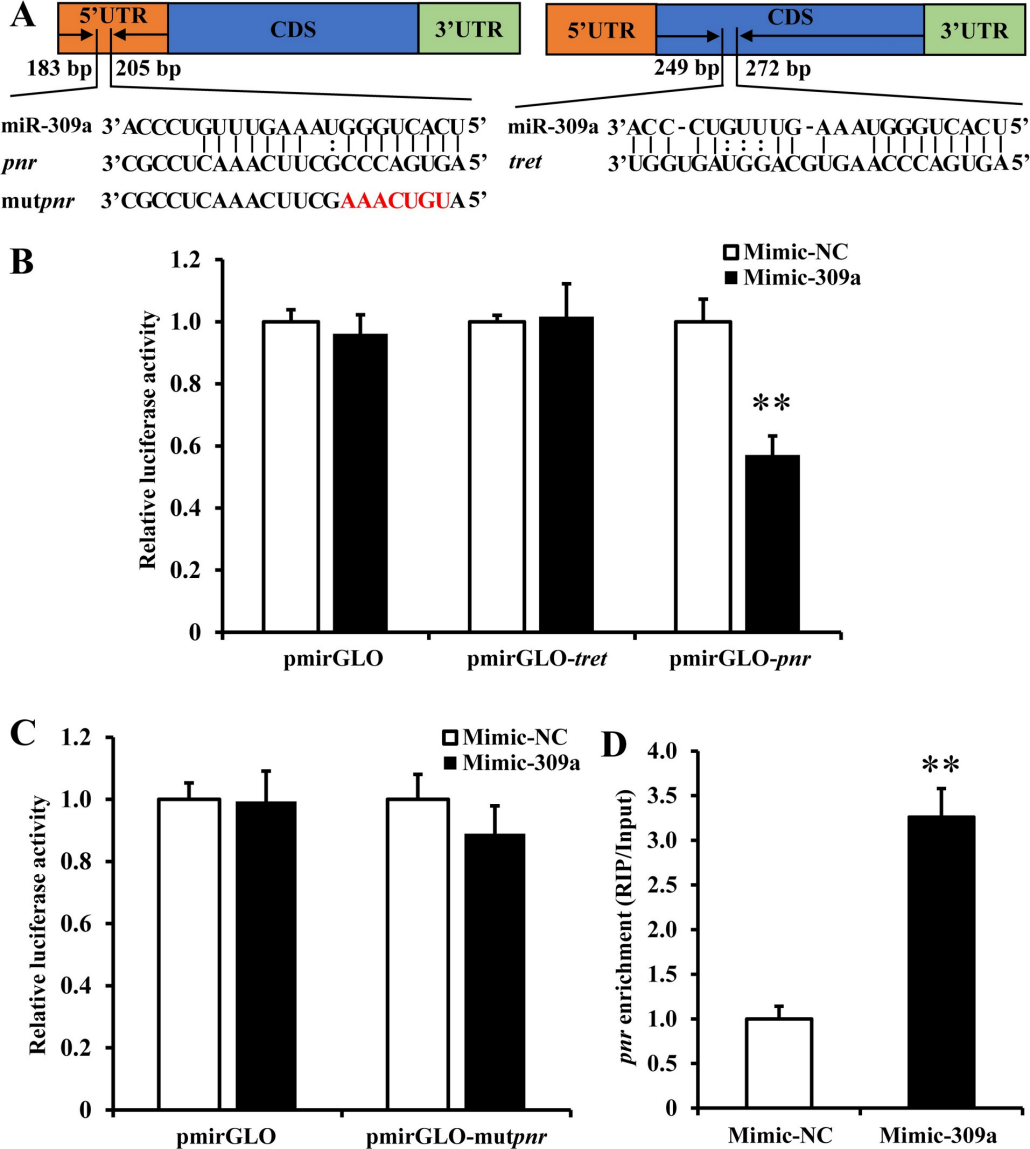
miR-309a targets *pnr* in *B*. *dorsalis*. (A) Binding region information for *pnr* and *tret*. (B) Dual luciferase reporter assays in wild types of *pnr* and *tret*. (C) Dual luciferase reporter assays in mutant *pnr* using HEK293T cells co-transfected with miRNA mimic and pmirGLO/recombinant pmirGLO vectors containing the predicted binding sites or mutant binding sites for *B* and *C*. Data are means ± SE (error bars) of four biological replications. (D) miR-309a targets *pnr in vivo* as demonstrated by RNA immunoprecipitation assay. Data are means ± SE of three biological replications. The differences between means were analyzed by Student’s *t* test for *B*, *C*, and *D*. For the significance test: unmarked * indicates not significant; **P < 0.01.

To assess the interaction between miR-309a and the two candidate targets (*pnr* and *tret*) *in vitro*, a dual-luciferase reporter assay was performed wherein HEK293T cells were co-transfected with mimic-309a and a recombinant pmirGLO vector containing either a 300 bp *pnr* gene 5’ UTR or *tret* gene CDS that contained the putative miR-309a binding site (Fig 4A). We observed a reduction (*P* = 0.011) in luciferase activity by 43% relative to the control in cells that were co-transfected with mimic-309a and pmirGLO-*pnr*. In contrast, no difference in luciferase activity was observed when cells were co-transfected with mimic-309a and pmirGLO-*tret* (Fig 4B). However, when cells were co-transfected with the mimic-309a and pmirGLO-mut*pnr* with a mutated miR-309a binding site, luciferase activity was recovered to a similar level as in the control (Fig 4C).

Once we confirmed *in vitro* that miR-309a interacts with *pnr*, an RNA immunoprecipitation (RIP) assay was performed to validate the binding status of miR-309a with *pnr* or *tret* RNA *in vivo*. The RIP results revealed that the abundance of *pnr* mRNA was significantly (*P* = 0.003) enhanced by 3.3-fold in the Ago-1 antibody immunoprecipitated RNAs from mimic-309a-injected ovaries compared to the mimic-NC control (Fig 4D). Moreover, the mRNA abundance of *tret* was not significantly changed in RIP assay, therefore the candidate target *tret* was finally excluded (S2C Fig). Collectively, these results strongly suggest that *pnr* is the authentic target of miR-309a, and miR-309a directly interacts with *pnr*.

### *Vg*-related genes targeted by *pnr* in the miR-309a regulatory pathway

**Fig 5:**
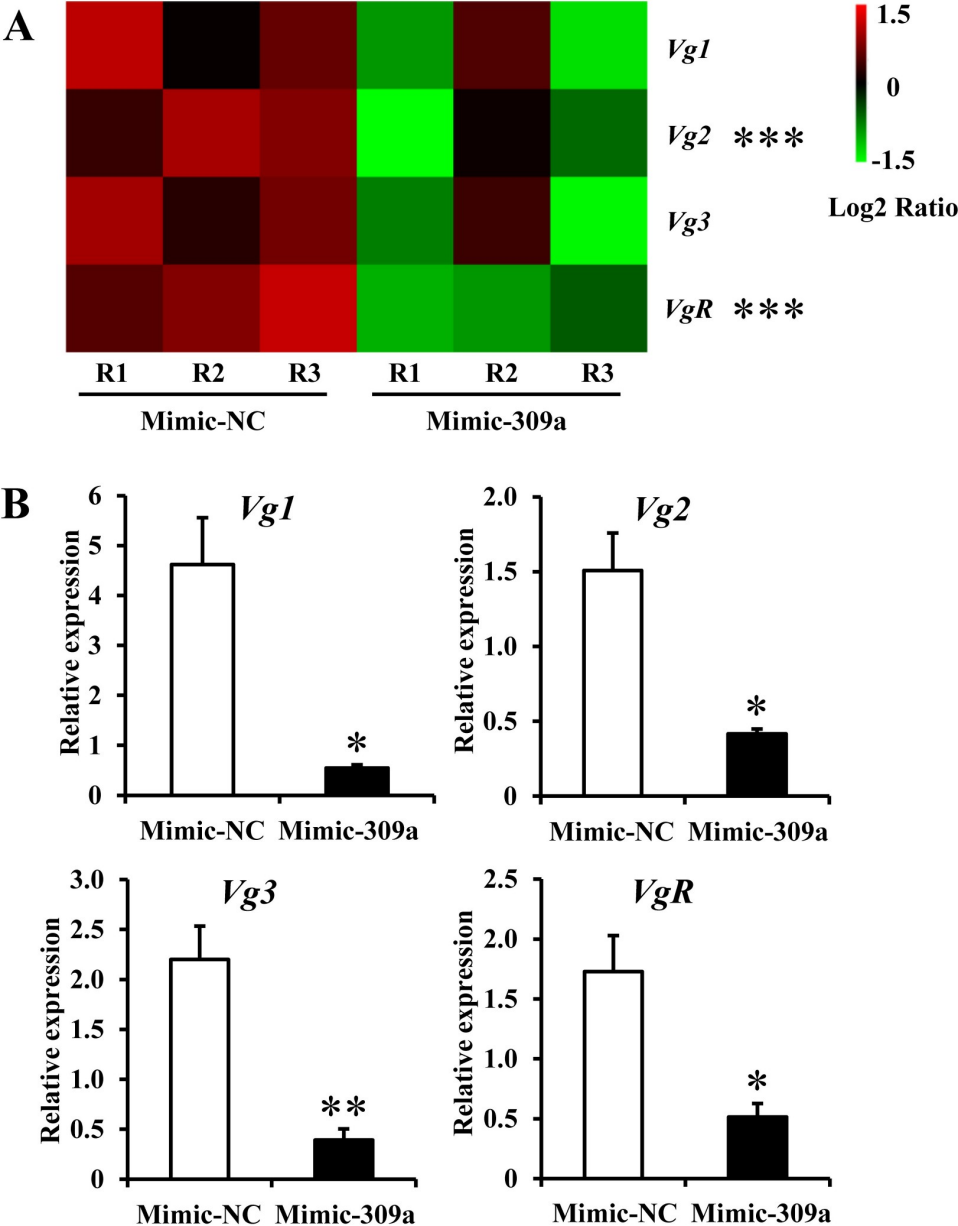
Relative expression of *Vg-*related genes (*Vg1*, *Vg2*, *Vg3*, and *VgR*) after mimic-309a injection. (A) Illumina sequencing results. For the significance test: unmarked * indicates not significant; ***false discovery rate (FDR, an adjusted *P*-value) < 0.001 with log_2_|Fold change| > 2. R1, R2, and R3 indicate three independent biological replications. The color code indicates the fold change of the gene abundance in the form of a logarithm. The fragments per kilobase of exon per million fragments mapped (FPKM) of genes is normalized in each row. (B) qPCR results. *a-tubulin* and *rps3* as reference genes were used to normalize the expression of *Vg-*related genes. The differences between means were analyzed by Student’s *t* test. For the significance test: *P < 0.05; **P < 0.01. Data are means ± SE (error bars) of four biological replications.

The *pnr* is a transcription factor, so we further investigated its interacting downstream genes. Firstly, we analyzed the relative expression level changes of genes involved in ovarian development via transcriptome sequencing of miR-309a-overexpressed female adults. We found that *Vg*-related genes *Vg1*, *Vg2*, *Vg3*, and *VgR* that directly affect ovarian development were repressed after miR-309a overexpression. qPCR used to analyze the relative expression levels of *Vg*-related genes after the overexpression of miR-309a showed a significant decrease (*P* = 0.022, 0.021, 0.002, and 0.025 for *Vg1*, *Vg2*, *Vg3*, and *VgR*, respectively) in the expression levels of these genes compared to the control. This observation was consistent with the Illumina RNA sequencing results and indicated that miR-309a mediates the expression of *Vg-*related genes in *B*. *dorsalis* (Fig 5 and S3 Data). Meanwhile, whether *Vg-*related genes are the interacting downstream genes of *pnr* attracted our attention.

**Fig 6:**
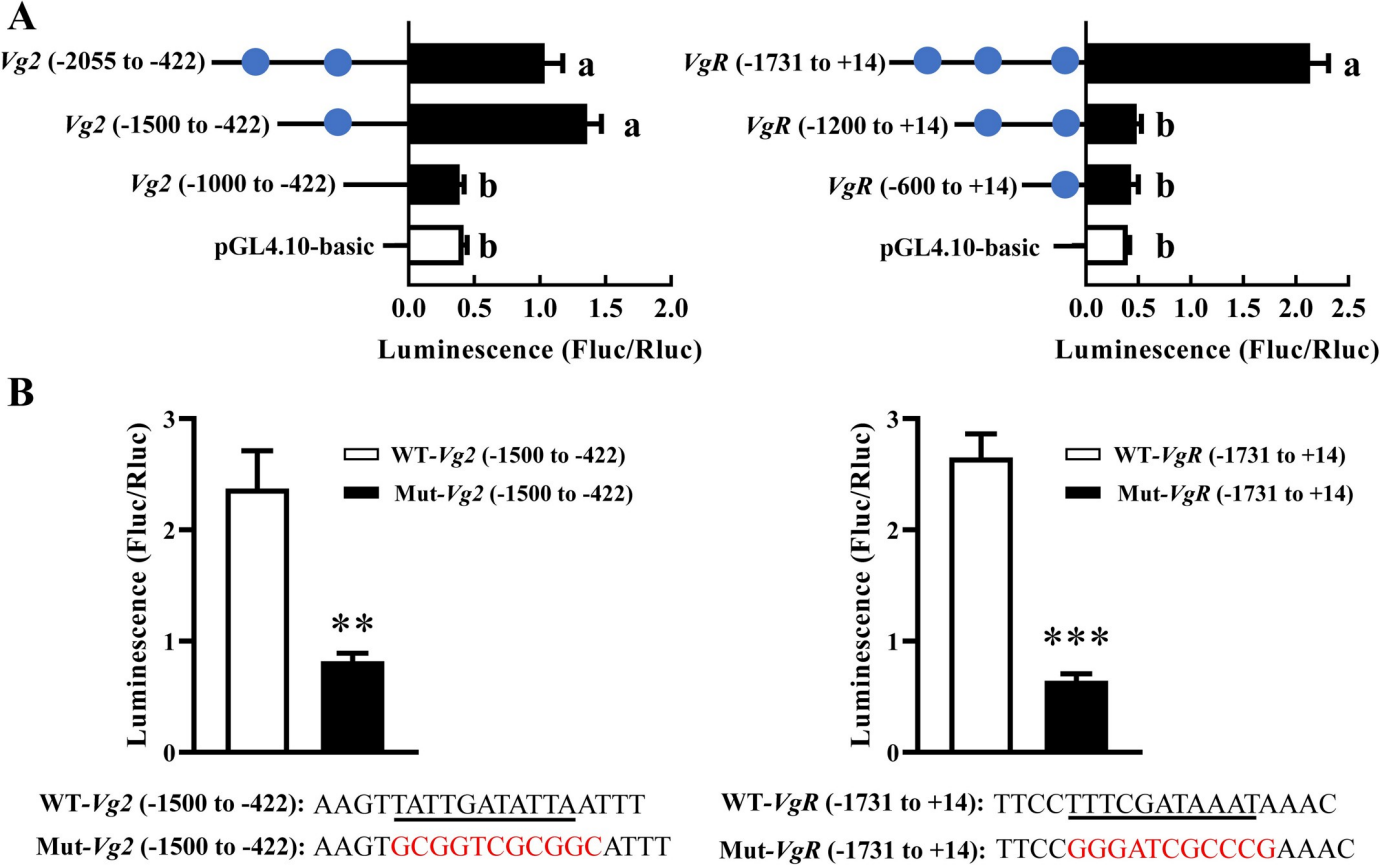
The luciferase activities in cells transfected with *Vg2*/*VgR* promoters and *pnr* expression construct. (A) pGL4.10-*Vg2* or pGL4.10-*VgR* promoter fragments and pGL4.74 were co-transfected into HEK293T cells with the transcription factors *pnr* in pcDNA3.1-EGFP. pGL4.10-basic vector was used as a control. A blue circle indicates a predicted binding site responding to *pnr*. (B) The luciferase activities of pGL4.10-*Vg2*^−1500 to –422^ or pGL4.10-*VgR*^–1731 to +14^ promoter with the mutant binding sites. Underlined sequence means the putative binding sites, and the sequence in read represents the mutant binding sites. Data are means ± SE (error bars) of four biological replications. The differences between means were analyzed by Student’s *t* test. For the significance test: **P < 0.01; ***P < 0.001. Different letters above the bars indicate significant differences among pGL4.10-*Vg2* or pGL4.10-*VgR* promoter fragments (Tukey HSD, ANOVA, *P* < 0.05).

To examine this query, we conducted *in silico* prediction analysis (JASPAR, https://jaspar.genereg.net/) where *pnr* interacted with *Vg*-related genes. This analysis predicted a potential regulatory relationship between *pnr* and *Vg1*, *Vg2*, *Vg3*, and *VgR* (Fig 6). To validate this prediction, we performed luciferase reporter assays to determine whether *pnr* could regulate luciferase activity by interacting with *Vg*-related gene promoters (*Vg1*^−1448 to +21^, *Vg2*^−2055 to –422^, *Vg3*^−1717 to –100^, *VgR*^–1731 to +14^). At first, the promoters were gradually truncated into 3 parts. We observed that the luciferase activity increased most (3.3-fold) in cells that was transfected with pGL4.10-*Vg2*^−1500 to –422^ promoter, and only increased (5.4-fold) when transfected with pGL4.10-*VgR*^–1731 to +14^ promoter, compared with the pGL4.10-basic vector, suggesting that the binding elements located between -1000 and -1500, -1200 and -1731 bp for *Vg2* and *VgR* promoter, respectively. Then, the only putative binding site in this region (*Vg2*: TATTGATATTA, *VgR*: TTTCGATAAAT) became the focus, and we mutated the nucleotide sequence of the putative binding site. The luciferase activity significantly decreased by 65% (*P* = 0.004) and 76% (*P* = 0.001) in cells that was transfected with the mutant pGL4.10-*Vg2*^−1500 to –422^ and pGL4.10-*VgR*^–1731 to +14^ promoter, relative to the normal promoter, respectively. No significant difference in luciferase activity was found when the cells were transfected with pGL4.10-*Vg1* or pGL4.10-*Vg3* promoter (S5 Fig). These results demonstrated that *pnr* interacted with *Vg2* and *VgR* by the binding site TATTGATATTA and TTTCGATAAAT, respectively.

### The miR-309a/*pnr* regulatory pathway is affected by upstream JH

**Fig 7:**
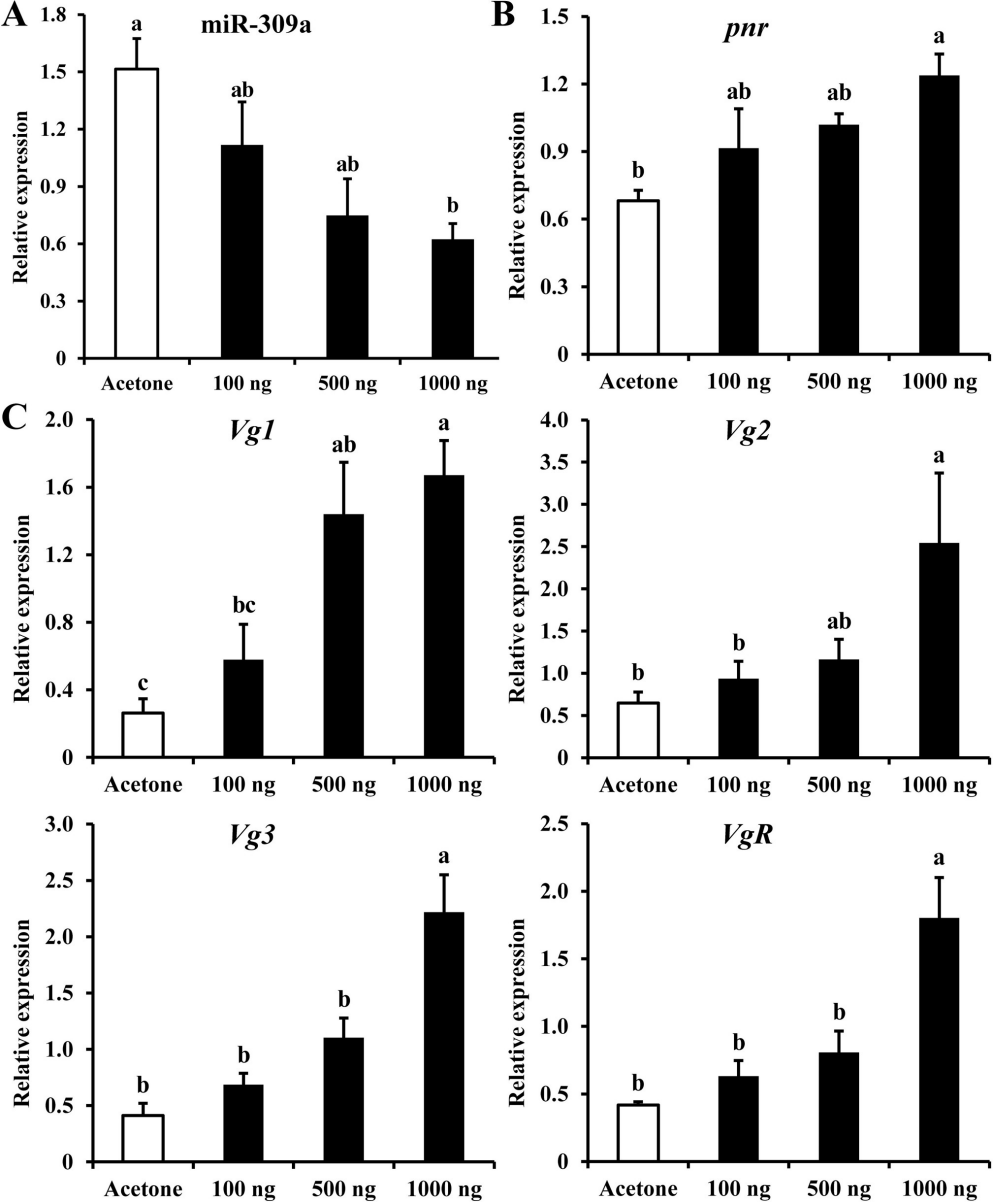
Effects of methoprene treatment on the expression of miR-309a regulatory pathway genes in *B*. *dorsalis* female adults. (A) Relative expression of miR-309a. (B) Relative expression of *pnr*. (C) Relative expression of *Vg*-related genes. Data are means ± SE (error bars) of four biological replications. U6 or *a-tubulin* and *rps3* were reference genes used to normalize the expression of miRNA or mRNA. Different letters above the bars indicate significant differences of the miRNA or mRNA at different doses of methoprene (Tukey’s HSD, ANOVA, *P* < 0.05).

During transcriptome analysis of DEGs in miR-309a-overexpressed female adults of *B*. *dorsalis*, the expression profiles of JH-related (*protein takeout-like*, *juvenile hormone epoxide hydrolase 2* and *uncharacterized protein*) and 20E-related (*cytochrome P450 307a1*, *cytochrome P450 315a1*, *ecdysone-induced protein 74EF* (*E74*) *and ecdysone 20-monooxygenase*) genes were significantly changed (S6 Fig). To clarify the roles of JH and 20E in the miR-309a regulatory pathway, the expression levels of miR-309a regulatory pathway genes were measured. Treatment of three-day-old female adults with JH-analog methoprene (1000, 500, and 100 ng/adult) decreased the expression level of miR-309a in a dose-dependent manner. With 1000 ng/adult of methoprene, miR-309a was down-regulated (59%, *P* < 0.05) (Fig 7A), while *pnr* was up-regulated (82%, *P* < 0.05) (Fig 7B). Similar to *pnr*, there was a significant increase in the expression of *Vg*-related genes with 1000 ng/adult methoprene treatment compared to the control; the respective expression levels of *Vg1*, *Vg2*, *Vg3*, and *VgR* were increased by 5-, 3-, 4-, and 4-fold (*P* < 0.05) (Fig 7C). In addition, we found that the expression level of primary transcript of miR-309a (pri-miR-309a) was significantly decreased (71%, *P* = 3 × 10^−4^) after the methoprene application at 1000 ng/adult (S7 Fig).

The application of 20E at 1000 ng/adult also resulted in significant up-regulation (*P* < 0.05) of the expression of *Vg*-related genes compared to the control (S8 Fig). However, miR-309a and *pnr* were not significantly changed (S8 Fig). Similarly, the expression level of pri-miR-309a was not significantly changed after the 20E treatment at 1000 ng/adult (S7 Fig).

**Fig 8:**
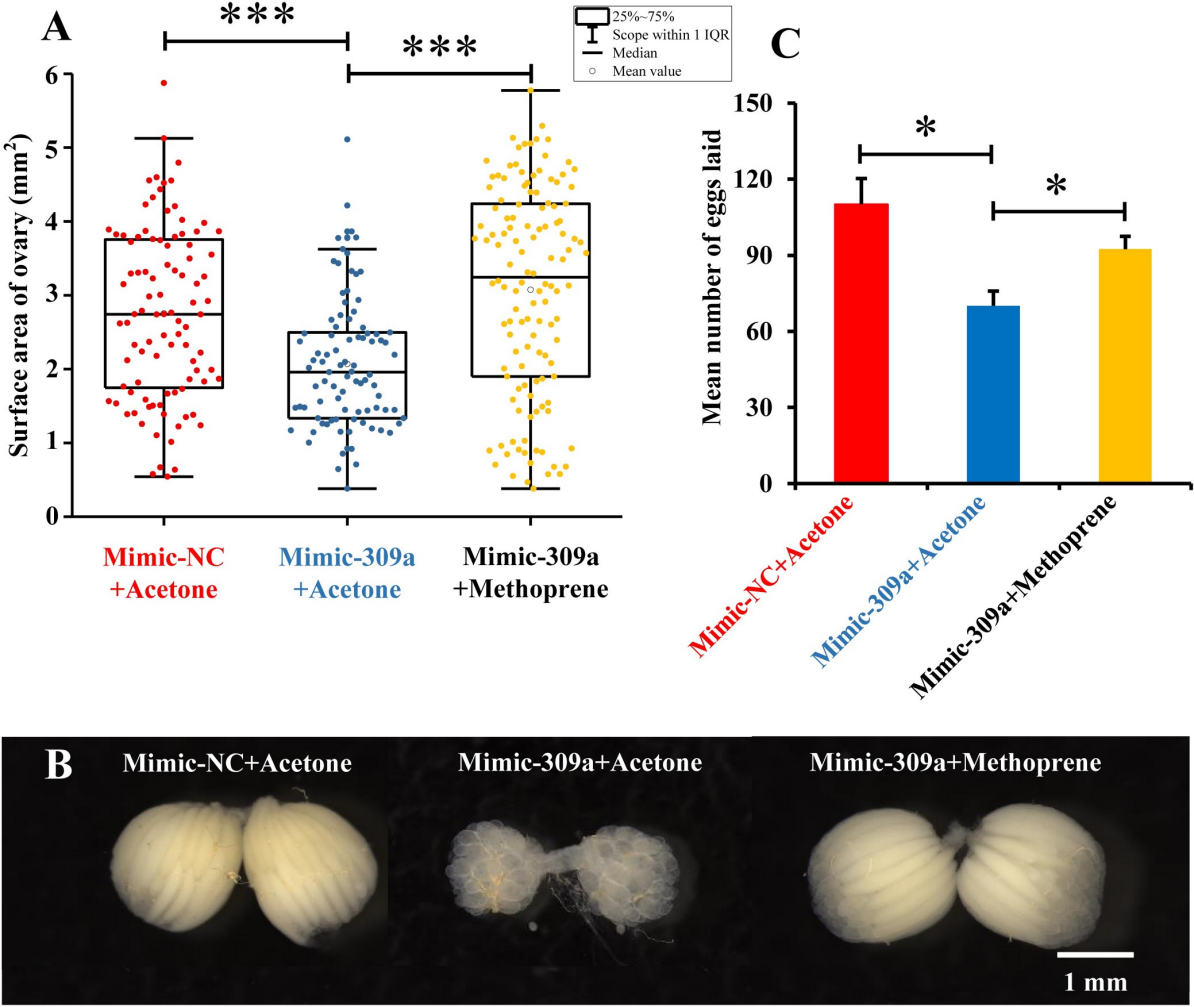
Effects of methoprene treatment on ovarian development in miR-309a overexpressed female adults of *B*. *dorsalis*. (A) Surface area of the ovary, including the mimic-NC-injected females with acetone treatment (mimic-NC+acetone, n = 96), mimic-309a-injected females with acetone treatment (mimic-309a+acetone, n = 91), and mimic-309a-injected females with methoprene treatment (mimic-309a+methoprene, n = 126). (B) Egg number. The fecundity of miR-309a-overexpressed females in methoprene treatment after successfully mating with untreated naive males. (C) Ovary phenotypes. Data are means ± SE (error bars) of three biological replications. The differences were analyzed by Student’s *t* test. For the significance test: *P < 0.05, ***P < 0.001.

Next, we performed a rescue experiment in which the three-day-old female adults were treated with mimic-309a to overexpress miR-309a, after which they were either treated with methoprene (1000 ng/adult) or an equal volume of acetone. The average surface area of ovaries in mimic-309a-treated females overexpressing miR-309a (2.07 mm^2^) was significantly smaller (*P* = 1 × 10^−5^) compared to the negative control of mimic-NC-treated females (2.75 mm^2^). However, when females overexpressing miR-309a were treated with methoprene, the average surface area of the ovaries recovered to about the same size (3.07 mm^2^) as in the negative control (*P* = 0.066), and these were also larger (*P* = 8.82 × 10^−10^) than for non-methoprene rescued females overexpressing miR-309a (Fig 8A). This indicated that the abnormal development of the ovary in females overexpressing miR-309a could be rescued by methoprene (Fig 8B). Furthermore, the average number of eggs laid by females overexpressing miR-309a (70 eggs/adult) decreased (*P* = 0.024) compared to the negative control of mimic-NC-treated females (110 eggs/adult). Also, the number of eggs laid by methoprene-rescued females (92 eggs/adult) increased significantly (*P* = 0.029) compared to non-methoprene-rescued females (Fig 8C).

## Discussion

Herein, we investigated how miR-309a regulates ovarian development and explored the factors that could be involved in its regulatory pathway. Using a combination of *in silico* prediction tools together with *in vitro* and *in vivo* assays, we found that miR-309a targets the gene *pnr* in *B*. *dorsalis*. Our findings indicate that miR-309a binds to the 5’ UTR of *pnr* to control its posttranscriptional regulation, with an increase in the expression of miR-309a being correlated with a decrease in the expression of *pnr* and vice versa. In another Dipteran, the yellow fever mosquito *A*. *aegypti*, miR-309 has been reported to regulate ovarian development by targeting the Homeobox gene *SIX4* [1]. In this study, we first searched for *SiX4* in *B*. *dorsalis*, and no *SIX4* ortholog was identified in *B*. *dorsalis*. Furthermore, we conducted a homology analysis of miR-309a in all species present in miRbase. Out of the 35 miRNAs identified in 18 other species with sequence homology to miR-309a, 15 species belonged to Diptera (S4 Data). Additionally, the entire miR-309a sequence was not conserved among the different species, but their miRNA sequences showed conserved seed regions, suggesting that these miRNAs may have similar regulatory functions (S9 Fig). This may explain why *A*. *aegypti* miR-309 targets *SIX4*, while *B*. *dorsalis* miR-309a controls *pnr* to regulate ovarian development. Similar results, that miRNAs with conserved seed regions and even with identical sequences may regulate different target genes in different species, have been reported in previous studies. For example, miR-14 regulates development and metamorphosis by targeting the *E75* and *EcR*-*B* in *B*. *mori*, while it targets *Spook* and *EcR* in *Chilo suppressalis* [19,20]. Also, miR-2 has been reported to target *Krüppel homolog1* (*Kr*-*h1*) in *Blattella germanica*, while it targets *Notch* in *L*. *migratoria* [10,21].

Following the overexpression of miR-309a in this study, we noticed significant reduction in the expression levels of *Vg*-related genes (*Vg1*, *Vg2*, *Vg3*, and *VgR*) and *pnr* in *B*. *dorsalis*. *In vitro* assays showed that the transcription factor *pnr* directly activated *Vg2* and *VgR* expression. In other insects, *pnr* has been reported to be involved in reproduction. In *N*. *lugens*, the knockdown of *pnr* resulted in a reduced expression of the *Vg* gene, leading to fewer offspring [22]. Furthermore, several studies have confirmed that *pnr* directly regulates the expression of *Vg* to control reproduction. For example, as a transcriptional activator, GATA regulates the expression level of *Vg* to mediate ovarian development in *A*. *aegypti* [17]. Similarly, GATAβ4 promotes *Vg* transcription and egg formation in *B*. *mori* [16], while GATA acts as a transcriptional activator of *Vg*, leading to the regulation of vitellogenesis and reproduction in the Asian longhorned tick *Haemaphysalis longicornis* [23]. Regrettably, we could not effectively knockdown the expression of *pnr* through RNAi to better evaluate its effect on the expression of *Vg*-related genes (S2D Fig). The availability of other tools such as CRISPR/Cas9 for genome editing in *B*. *dorsalis* [24,25] could be exploited to achieve this in future experiments. Nevertheless, our results indicate that miR-309a targets *pnr* to regulate *Vg*-related genes, which in turn directly affects ovarian development in *B*. *dorsalis*.

JH and 20E are the most important hormones in insect development and reproduction, and they modify expression profiles of many miRNAs. Following treatment with 20E, the expression levels of *D*. *melanogaster* miR-252-3p [26], *N*. *lugens* miR-34 [27], miR-8-5p, and miR-2a-3p [28], and *B*. *mori* miR-281 [29] were significantly reduced, while *D*. *melanogaster* let-7 cluster (let-7/miR-100/miR-125) [30] and *B*. *dorsalis* let-7 [31] were significantly increased. Similarly, after treatment with JH, the expression levels of the *L*. *migratoria* miR-2 cluster (miR-2/miR-13a/miR-13b/miR-71) [10], let-7, and miR-278 [11] were significantly reduced, while *N*. *lugens* miR-34 [27] was significantly increased. In our study, the expression levels of JH- and 20E-related genes were significantly altered in miR-309a-overexpressed female adults, indicating that JH and 20E are potential upstream regulatory factors involved in the miR-309a regulatory pathway. The application of the JH analog methoprene resulted in the down-regulation of pir-miR-309a and miR-309a and contrasting up-regulation of *pnr* as well as *Vg*-related genes, while the application of 20E only resulted in the up-regulation of *Vg*-related genes. These results indicate that JH could mediate the miR-309a regulatory pathway in *B*. *dorsalis* via regulating the expression of miR-309a to in turn control the expression of downstream genes involved in ovarian development. Moreover, treatment of females overexpressing miR-309a with methoprene could rescue abnormal ovarian development, and this also led to recovery in egg production. However, miR-309a mimic is a double stranded RNA that function as processed/mature miR-309a and possess no transcriptional regulatory elements for JH pathway genes (e.g. JH receptor or transcriptional coregulators) to act on, demonstrating JH could directly upregulate the *Vg*-related genes to counteract the negative effects from the mimic. In a previous study, administration of a drop of methoprene on newly emerged fruit flies would up-regulate the expression levels of *Vg1* and *Vg2*, and accelerate the ovarian development in *B*. *dorsalis* [32], whereas miR-309a has no expression in the newly emerged female adults. These results indicated that JH could independently regulate the expression of *Vg*-related genes in the presence or absence of miR-309a. Although the mechanism through which 20E regulates miRNAs in insects is well understood, how JH regulates miRNAs is still largely unexplored. In *D*. *melanogaster*, 20E is known to induce the expression of the let-7 cluster (let-7-C) through its receptor *EcR*/*Ultraspiracle*, miR-252 through the 20E signaling pathway gene *broad-complex core protein* (*Br*-*C*), combined with the responsive element in the promoter region of the pri-let-7-C and pri-miR-252, while it suppresses the expression of miR-8 through the 20E pathway genes *EcR*, *Br*-*C*, or *E74* combined with the responsive element in the promoter region of the pri-miR-8 [26,30,33]. In *A*. *aegypti*, before blood feeding, 20E with a low titer represses miR-275 and miR-305 expression by *EcR* with the corepressor *SMRTER*, but a high titer of 20E activates their expression by *EcR* with the coactivator *Taiman* after a blood meal [34]. Additionally, JH/Met hierarchical network governs *A*. *aegypti* miR-2940 expression via *E75* to positively regulate the pri-miR-2940 [35]. Therefore, we speculate that JH regulates miR-309a through its pathway genes such as *Met*/*Taiman*, *Kr*-*h1* and *Hairy*. However, further studies will be required to confirm this hypothesis.

**Fig 9:**
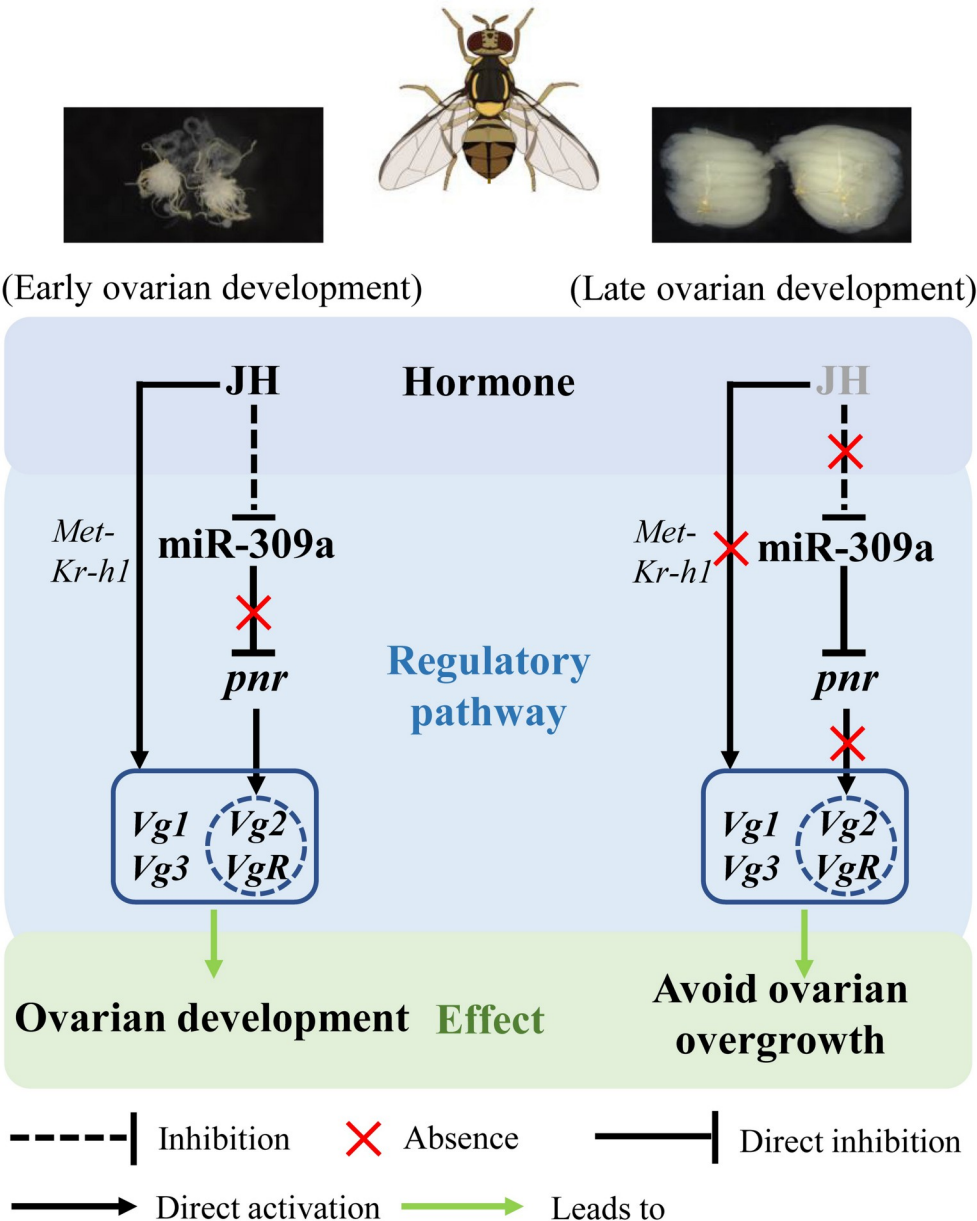
Mechanism of miR-309a in the regulation of ovarian development in *B*. *dorsalis*. During early ovarian development, miR-309a has lowered expression due to JH suppression and cannot inhibit *pnr* expression. At this stage, *pnr* induces the expression of *Vg*-related genes in ovarian development to maintain normal physiological activities. With the reduction of JH inhibition, miR-309a up-regulates and inhibits the expression of *pnr* to avoid excessive expression of *Vg*-related genes (*Vg2* and *VgR*). JH may act as the upstream switch of the miR-309a regulatory pathway to maintain the balance and stability of the whole pathway by inhibiting miR-309a expression.

In summary, we found that the microRNA miR-309a is an important regulator of ovarian development in *B*. *dorsalis*. In *A*. *aegypti* mosquitoes, JH titers were substantially elevated after eclosion, while blood feeding triggers a decline of JH [36–38]. Therefore, under normal conditions during early ovarian development in *B*. *dorsalis*, miR-309a is expressed at low levels by JH suppression and does not inhibit the expression of *pnr*. As a result, *pnr* induces the expression of *Vg*-related genes (*Vg2* and *VgR*). However, during late ovarian development, the expression of miR-309a is up-regulated due to the reduction of JH titer and in turn inhibits the expression of *pnr* to avoid excessive expression of *Vg*-related genes. JH may act as the upstream switch of the miR-309a regulatory pathway and maintains the balance and stability of the whole pathway by inhibiting the expression of miR-309a (Fig 9). Thus, a JH-controlled miR-309a/*pnr* regulatory axis has been identified in the ovarian development of Dipterans.

## Conclusions

Overall, we uncovered that miR-309a regulates the development of the ovary in an important dipteran pest, *B*. *dorsalis*, through a transcriptional factor, *pnr*. The uncovering of this JH-controlled miR-309a/*pnr* regulatory axis advances our understanding of the molecular mechanisms that regulate insect ovarian development. The findings in this study also provide new insights for potential targets to control arthropod pests through the reproductive strategy.

## Methods

### Insect rearing and total RNA extraction

The laboratory stock of *B*. *dorsalis* was cultured in an incubator under the following conditions: 27±0.5°C, 14:10 h (L:D) photoperiod, and 75±5% relative humidity. Larvae and adults were reared on artificial diets as previously described [39]. Total RNA was extracted with Trizol (Invitrogen, Carlsbad, CA) following the manufacturer’s instructions. The quality, concentration, and purity of the extracted RNA were measured by a NanoDrop One microvolume UV-Vis spectrophotometer (Thermo Scientific, Waltham, MA), and RNA integrity was checked by 1.0% agarose gel electrophoresis.

### qPCR

The synthesis of cDNA from miRNA and mRNA and quantitative analysis were conducted as previously described [40]. Briefly, for the miRNA assay, RNA samples were first polyadenylated with poly(A) polymerase and then reverse transcribed using a miRNA cDNA Synthesis Kit (CoWin Biosciences, Beijing, China). The poly(A)-tailed cDNA was subsequently amplified using a miRNA qPCR Assay Kit (CoWin Biosciences). For the mRNA and pri-miRNA assay [34], the cDNA was synthesized using a PrimeScript RT Reagent Kit (Takara, Dalian, China). Gene expression levels were assessed using the NovoStart SYBR qPCR SuperMix kit (Novoprotein, Shanghai, China). qPCR was performed on a CFX Connect Real-Time System (Bio-Rad, Hercules, CA). A list of all of the primers used in the assays is presented in S1 Table. The relative expression of miRNA was normalized using U6 as an internal reference, while for mRNA, we used *a-tubulin* (GenBank Accession No. GU269902) and *rps3* (XM_011212815) as internal references via the qBASE software (Biogazelle, Zwijnaarde, Belgium).

### Spatiotemporal expression analysis

To evaluate the temporal expression of miR-309a, *pnr*, *tret*, and *Vg*-related genes (*Vg1*, *Vg2*, *Vg3*, and *VgR*), female adults were collected within 1 h post-eclosion, and their ovaries were successively dissected and collected from the 1^st^ day to the 10^th^ day post-eclosion (recorded as A0–A10, respectively). Four biological replicates were performed, and each replicate contained 40 ovaries for A0–A2, 35 for A3 and A4, 30 for A5, 20 for A6, and 10 for A7–A10. The relative expression levels of the targets were analyzed by qPCR as described above.

Six-day-old female adults were selected (based on ovarian development stage) for dissection and evaluation of the expression of miR-309a in different tissues. The tissues used were the Malpighian tubules, gut, fat body, ovary, and remainder (the remaining tissues). Four biological replicates were performed, and each replicate contained 20 tissues.

### Fluorescence *in situ* hybridization

The Cy3 labeled probe was synthesized by GefanBio (Shanghai, China) for detecting miR-309a. A probe of *Caenorhabditis elegans* miRNA (UUGUACUACACAAAAGUACUG) was used as the negative control. The ovaries were dissected from seven-day-old female adults and fixed in 4% paraformaldehyde overnight at 4°C. The samples were treated with 0.25% hydrochloric acid and proteinase K, and then prehybridized for 1 h. Subsequently, these samples were incubated with the probes at 65°C for 48 h, and then washed five times in PBS. Nucleus were stained with DAPI at 500 times dilution. Fluorescent signals were captured by Zeiss LSM 780 confocal microscope (Zeiss, Jena, Germany).

### miR-309a mimic and inhibitor assay

According to the temporal expression pattern of miR-309a in the ovaries, we designed miR-309a mimic and inhibitor assays to evaluate the consequence of miR-309a overexpression or repression on reproductive processes such as ovarian development and oviposition. In line with this objective, we selected three-day-old females (representing a time point much earlier than the peak expression of miR-309a) and four-day-old females (representing a time point close to the peak expression) for injection with either 50 pmol (125 μM, 400 nL) of mimic-309a (Ribobio, Guangzhou, China) or antago-309a into the ventral abdomen of females using a Nanoliter 2010 injector system (WPI, Sarasota, FL). In a similar setup, an equivalent dose of mimic-NC or antago-NC was injected as the control. After 24 h of treatment, four individuals were randomly selected to evaluate the relative expression level of miR-309a by qPCR. Each treatment was performed with four biological replicates.

Based on the expression results for miR-309a, following the treatment with mimic/antagomir, we repeated the miR-309a overexpression assay. This time, we carefully checked for phenotypic changes in the ovary, following the injection of three-day-old female adults with mimic-309a. At four days post-treatment, seven-day-old female adults were dissected, and the morphology of the ovary (i.e., the surface area) was observed and recorded using a Leica M205A stereomicroscope (Leica Microsystems, Wetzlar, Germany) as previously described [32]. At six days post-treatment, nine-day-old female adults were mated with same age unmated males. After exposure for mating, the female adults that successfully mated (mating time longer than half an hour by observation) were reared alone, and the number of eggs laid within three days was counted [41]. Each treatment was performed with three biological replicates; each replicate contained 15 pairs.

### Transcriptome sequencing

To identify the target gene(s) of miR-309a in *B*. *dorsalis*, transcriptomic expression profiling of female adults in response to miR-309a mimic overexpression was performed. Three-day-old female adults were injected with 50 pmol of miR-309a mimic. After 24 h of treatment, 10 individuals were randomly selected to extract the total RNA. Each treatment was performed with three biological replicates. The RNA purity and concentration were measured using a NanoDrop 2000 Spectrophotometer (Thermo Scientific). The RNA integrity was assessed using the RNA Nano 6000 assay kit for the Agilent Bioanalyzer 2100 system (Agilent Technologies, Santa Clara, CA). A total of 1.5-μg RNA per sample was used as the input material for rRNA removal using the Ribo-Zero rRNA Removal Kit (Epicentre, Madison, WI). The cDNA library was sequenced on an Illumina Hiseq platform using a paired-end strategy. Transcriptome assembly and RNA-Seq analyses used the same strategy as in our previous study [42]. An absolute value of log_2_|Fold change| > 2 with false discovery rate ≤ 0.05 (FDR, an adjusted *P*-value) were considered as indicating significantly altered gene expression.

### *In silico* prediction and analysis of potential miR-309a target genes

Based on the miR-309a mature sequence and genome database (NCBI Assembly: ASM78921v2), four different software programs, PITA [43], qTar (https://github.com/zhuqianhua/qTar), miRanda [44], and RNAhybrid [45], were used with their default parameters to predict potential miR-309a binding sites in the predicted target genes. Bioassays were then performed as described above. After 24-h treatment with mimic-309a, four individuals were randomly selected to detect the relative expression levels of the predicted target mRNAs by qPCR. Each treatment was performed with four biological replicates.

### Dual-luciferase reporter assay: *In vitro* verification of predicted targets

An ~300 bp 5’ untranslated region (5’ UTR) and coding sequence (CDS) from the predicted miR-309a target sites in *pnr* and *tret*, respectively, were separately cloned into the pmirGLO vector (Promega, Madison, WI), downstream (pmirGLO-*pnr* and pmirGLO-*tret*) of the luciferase gene using the SacI and XhoI restriction sites. Based on the results from the dual luciferase assay, we constructed a mutant sequence for *pnr* mutation, where 7 bp of the binding site corresponding to the seed region of miR-309a were mutated to obtain the pmirGLO-mut*pnr* using the QuickMutation kit (Beyotime, Beijing, China) according to the manufacturer’s instructions. The constructed vectors, miR-309a mimic, or mimic-NC were transferred into HEK293T cells using the TransIT-LT1 transfection Reagent (Mirus Bio, Madison, WI) according to the manufacturer’s instructions. The activities of the Firefly and Renilla luciferases were measured after 24 h of transfection with the Dual-Glo Luciferase Assay System (Promega, Madison, WA) using a TriStar2 LB 942 multimode microplate reader (Berthold Technologies, Bad Wildbad, Germany).

### RIP: *In vivo* verification of predicted targets

A RIP experiment was performed using a Magna RIP kit (Millipore, Billerica, MA) according to the manufacturer’s instructions. Three-day-old female adults were injected with 50 pmol of miR-309a mimic, and mimic-NC was injected as the control. After 24 h, the treated adults were subjected to RIP analysis. Four individuals were collected and homogenized in ice-cold RIP lysis buffer. The homogenates were stored at −80°C overnight. A total of 5 μg of Ago-1 antibody (Abcam, Cambridge, UK) or normal mouse IgG (Millipore) was pre-incubated with magnetic beads. The frozen homogenates in the RIP lysates were thawed and centrifuged, and the supernatants were incubated with the magnetic bead–antibody complex at 4°C overnight. The immunoprecipitated RNAs were reverse-transcribed into cDNA using random hexamers. qPCR was performed to quantify *pnr*. The supernatants of the RIP lysates (input) and the IgG controls were assayed to normalize the relative expression levels of the target gene [46].

### Analysis of the expression of *Vg*-related genes following the overexpression of miR-309a

Three-day-old female adults were injected with 50 pmol of miR-309a mimic. After 24 h of treatment, four individuals were randomly selected to detect the relative expression level of *Vg***-**related genes (*Vg1*, *Vg2*, *Vg3*, and *VgR*) by qPCR; four biological replicates were used for each treatment.

### Dual-luciferase reporter assay–*in vitro* verification of regulatory relationship between *pnr* and *Vg*-related genes

To further understand whether *pnr* could regulate the expression of *Vg*-related genes, a 2000 bp fragment of the 5’-flanking regions of *Vg1*, *Vg2*, *Vg3*, and *VgR* as a putative promoter were predicted and analyzed using online software [47]. The 5’-flanking regions of *Vg1*, *Vg2*, *Vg3*, and *VgR* containing all *pnr* putative binding sites were amplified from *B*. *dorsalis* genomic DNA extracted from female adults (seven-day-old) using a DNeasy Blood & Tissue Kit (Qiagen, Hilden, Germany) by PCR and cloned into the pGL4.10 reporter plasmids (Promega) containing the firefly luciferase gene to obtain the pGL4.10-*Vg1*^−1448 to +21^, -*Vg2*^−2055 to –422^, -*Vg3*^−1717 to –100^, and -*VgR*^–1731 to +14^. The CDS of *pnr* was cloned into the pcDNA3.1-EGFP expression vector (Invitrogen) to obtain the pcDNA3.1-EGFP-*pnr*. The pGL4.10 constructed vectors (200 ng), a reference reporter pGL4.74 plasmid (Promega, 100 ng, containing the hRluc reporter gene), and the pcDNA3.1-EGFP-*pnr* or pcDNA3.1-EGFP (200 ng) were transferred into HEK293T cells (24-well plates) using the TransIT-LT1 transfection Reagent (Mirus Bio) according to the manufacturer’s instructions. At 48 h post-transfection, the activities of the Firefly and Renilla luciferases were measured as described above. The primers used for the construction of reporter plasmids are listed in S1 Table.

### dsRNA synthesis and RNAi assay

The dsRNA (ds*pnr* and ds*GFP*) was transcribed by a Transcript Aid T7 High Yield Kit (Thermo Scientific, Wilmington, DE) according to the manufacturer’s instructions. Subsequently, 2 μg of ds*pnr* or ds*GFP* (control) was injected into the ventral abdomen of three-day-old female adults. At 24 and 48 h post-treatment, four individuals were randomly selected to detect the relative expression level of *pnr* by qPCR, and each treatment was performed with four biological replicates.

### Evaluation of the effects of JH analog and 20E on the miR-309a regulatory mechanism

To evaluate the effects of hormones on the expression of miR-309a, *pnr*, and *Vg*-related genes, we used a JH-analog (methoprene) and 20E (both from Sigma, Saint-Louis, MO). Methoprene and 20E were first dissolved in acetone and alcohol, respectively, and diluted to three final concentrations of 1000, 500, and 100 ng/μL. Then, the pronotum of three-day-old female adults was treated by applying a drop (1 μL) of the prepared hormone solutions, and acetone and alcohol were used as controls. At 24 h post-treatment, four individuals were randomly selected, and the relative expression levels of miR-309a pathway genes were detected by qPCR. Each treatment was performed with four biological replicates.

Additionally, rescue experiments using methoprene following treatment with mimic-309a to overexpress miR-309a were performed. First, three-day-old female adults were injected with 50 pmol of mimic-309a; at 24 h post-treatment, 1 μL of methoprene (1000 ng/μL) was applied, and acetone was used as a control. The morphology and surface area of the ovary and the number of eggs laid were recorded as described above.

### Homology analysis

To determine whether miR-309a regulates *B*. *dorsalis* ovarian development via the same target gene, i.e., the Homeobox gene *SIX4* as in *A*. *aegypti*, we conducted a homology analysis of *SIX4* and miR-309a. The *A*. *aegypti* Homeobox gene *SIX4* sequence (GenBank Accession No: AAEL010327-PA) was used as a query to BLAST search for its ortholog in the genome (NCBI Assembly: ASM78921v2) of *B*. *dorsalis* using the GenBank, NCBI, with default parameters. Furthermore, the mature sequence of miR-309a in *B*. *dorsalis* was used as a query to BLAST against the miRNA database miRBase (V 22.1) with default parameters. All miRNAs with homology to miR-309a were recorded. The miRNA sequence with the lowest E-value from each of the species was selected and used in a multiple alignment analysis to further evaluate sequence homology with miR-309a.

### Statistical analysis

Statistical analyses were performed using the SPSS 23.0 software (SPSS, Chicago, IL). Prior to analysis, Kolmogorov-Smirnov or Shapiro-Wilk tests were used to test for normality of all of the data. One-way analysis of variance (ANOVA) followed by Tukey’s HSD post-hoc test (*P* < 0.05) was used for multiple comparisons, and Student’s *t* test (*P < 0.05; **P < 0.01; ***P < 0.001; ns, not significant) was used for pairwise comparisons.

## Supporting information

S1 Fig Localization of miR-309a in the ovary of *B*. *dorsalis* by miRNA fluorescence in situ hybridization. The blue and red signals are DAPI and miR-309a.
(TIF)

S2 Fig Relative expression of miRNA and mRNA. (A) Relative expression of miR-309a at 24 h after the antago-309a injection, at 48 h after the mimic-309a or antago-309a injection. (B) Relative expression of *pnr* and *tret* at 24 h after the antago-309a injection. (C) Relative expression of *tret* at 24 h after the mimic-309a injection in RNA immunoprecipitation assay. (D) Relative expression of *pnr* at 24 and 48 h after ds*pnr* injection. Data are means ± SE (error bars) of four biological replications. U6 or *a*-*tubulin* and *rps3* were the reference genes used to normalize the expression of miRNA or mRNA. The differences between means were analyzed by Student’s *t* test. For the significance test: unmarked * indicates not significant.
(TIF)

S3 Fig Potential targets of miR-309a by co-analysis with prediction software programs. (TIF)

S4 Fig Relative expression of nine predicted target genes after miR-309a overexpression. *a-tubulin* and *rps3* were the reference genes used to normalize the expression of mRNA. The differences between means were analyzed by Student’s t test. For the significance test: unmarked * indicates not significant.
(TIF)

S5 Fig The luciferase activities in cells transfected with *Vg1*/*Vg3* promoters and *pnr* expression construct. pGL4.10-*Vg1*^−1448 to +21^ or pGL4.10-*Vg3*^−1717 to –100^ promoter fragments and pGL4.74 were co-transfected into HEK293T cells with the transcription factor *pnr* in pcDNA3.1-EGFP. pGL4.10-basic vector was used as a control. A blue circle indicates a predicted binding site responding to *pnr*. Data are means ± SE (error bars) of four biological replications. Same letters above the bars indicate no significant difference among pGL4.10-*Vg1* or pGL4.10-*Vg3* promoter fragments (Tukey HSD, ANOVA, *P* < 0.05).
(TIF)

S6 Fig Differentially expressed JH-related and 20E-related genes after mimic-309a injection via Illumina sequencing. (A) JH-related genes, including significantly down-regulated *protein takeout-like* (LOC105228734 and LOC105234181), *juvenile hormone epoxide hydrolase 2* (LOC105230541), *juvenile hormone epoxide hydrolase 2-like* (LOC105230714), and *uncharacterized protein* with juvenile hormone binding protein domain (LOC105232270, LOC105232271, LOC105232426, and LOC109579562) and significantly up-regulated *protein takeout-like* (LOC105226548). (B) 20E-related genes, including significantly down-regulated *cytochrome P450 315a1*, *mitochondrial* (LOC105232707), *cytochrome P450 307a1-like* (LOC105225301), and *ecdysone 20-monooxygenase* (LOC105225924) and significantly up-regulated *ecdysone-induced protein 74EF* (LOC105225270). For the significance test: false discovery rate (FDR, an adjusted *P*-value) < 0.001 with log_2_|Fold change| > 2. R1, R2, and R3 indicate three independent biological replications. The color code indicates the fold change of the gene abundance in the form of a logarithm. The fragments per kilobase of exon per million fragments mapped (FPKM) values of genes are normalized in each row.
(TIF)

S7 Fig Effects of methoprene and 20E treatment at 1000 ng/adult on the expression of pri-miR-309a in *B*. *dorsalis* female adults. Data are means ± SE (error bars) of four biological replications. *a*-*tubulin* and *rps3* were the reference genes used to normalize the expression of pri-miR-309a. The differences between means were analyzed by Student’s *t* test. For the significance test: unmarked * indicates not significant; ***P < 0.001.
(TIF)

S8 Fig Effects of 20E treatment on the expression of miR-309a regulatory pathway genes in *B*. *dorsalis* female adults. (A) Relative expression of miR-309a. (B) Relative expression of *pnr*. (C) Relative expression of *Vg*-related genes. Data are means ± SE (error bars) of four biological replications. U6 or *a*-*tubulin* and *rps3* were the reference genes used to normalize the expression of miRNA or mRNA. Different letters above the bars indicate significant differences of the miRNA or mRNA at different 20E doses (Tukey’s HSD, ANOVA, *P* < 0.05).
(TIF)

S9 Fig Homology analysis of *B*. *dorsalis* miR-309a. The homology analysis was conducted by blasting in the miRBase database. E indicates E-value.
(TIF)

S1 Table Primer sequences used in this study. (DOCX)

S1 Data Differentially expressed genes in miR-309a-overexpressed female adults. The absolute value of log_2_|Fold change| > 2 with false discovery rate ≤ 0.05 (FDR, an adjusted *P*-value) was considered as genes with significantly altered expression.
(XLSX)

S2 Data The information of 11 predicted target genes. (XLSX)

S3 Data *Vg*-related genes in miR-309a-overexpressed female adults. (XLSX)

S4 Data miRNAs in other species sharing sequence homology with *B*. *dorsalis* miR-309a. (XLSX)
