# Supplementary figures and images for: miR-309a is a regulator of ovarian development in the oriental fruit fly Bactrocera dorsalis
Source: PLoS Genet. 2022 Sep 16;18(9):e1010411. doi: 10.1371/journal.pgen.1010411 (PMC9518882; doi:10.1371/journal.pgen.1010411)

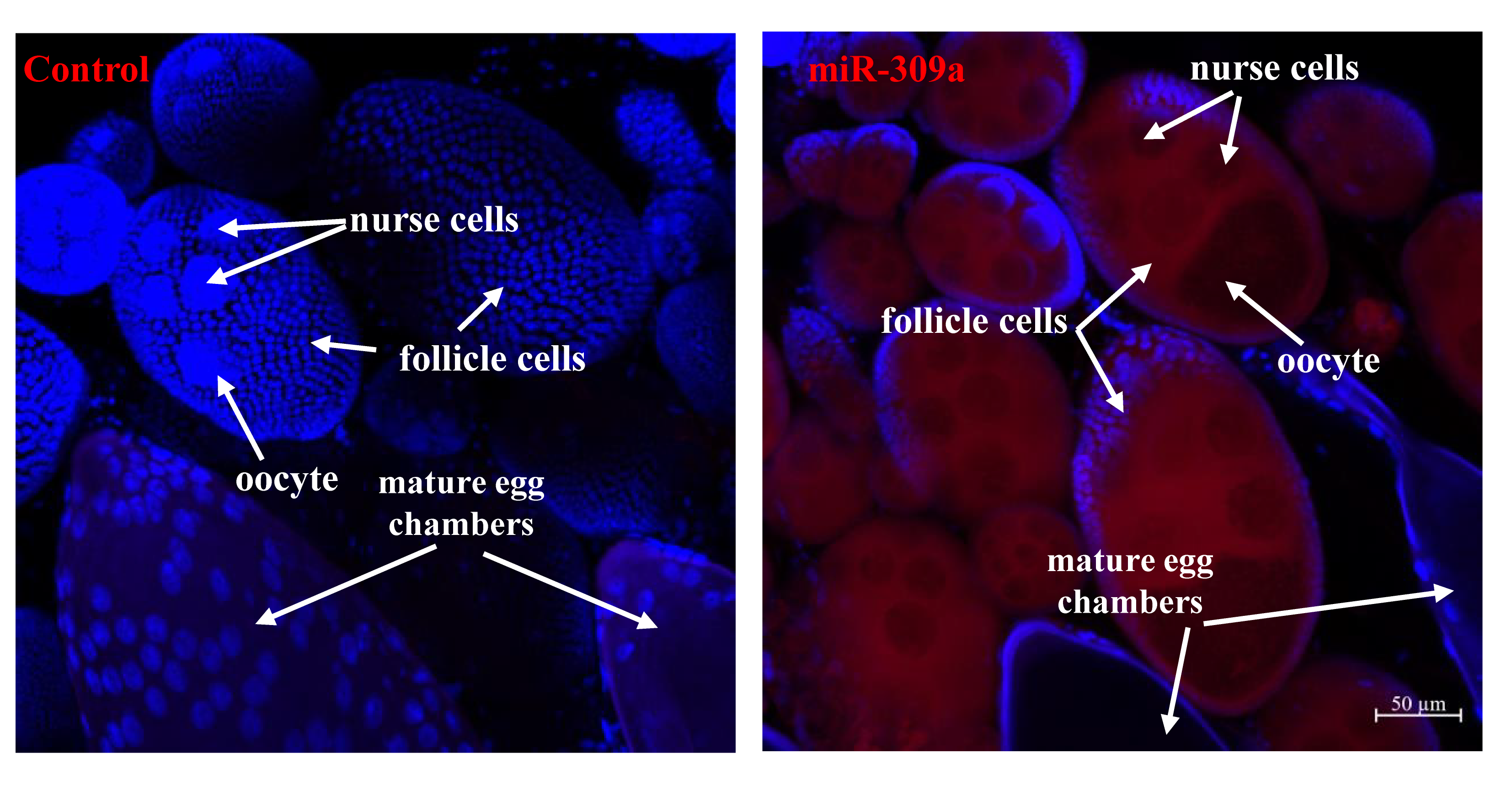

Supplement: S1 Fig — The blue and red signals are DAPI and miR-309a. (TIF) [file pgen.1010411.s001.tif]

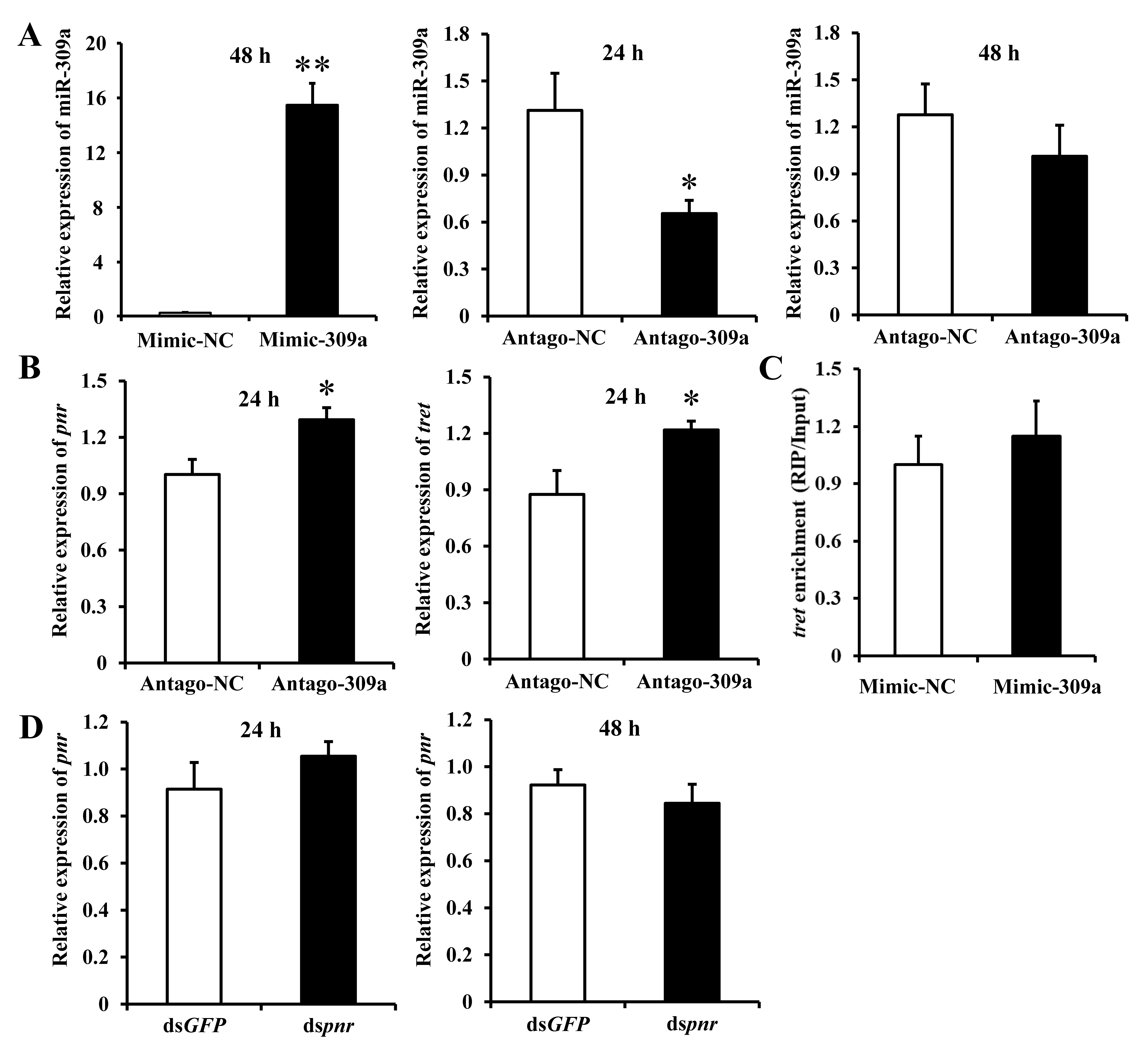

Supplement: S2 Fig — (A) Relative expression of miR-309a at 24 h after the antago-309a injection, at 48 h after the mimic-309a or antago-309a injection. (B) Relative expression of pnr and tret at 24 h after the antago-309a injection. (C) Relative expression of tret at 24 h after the mimic-309a injection in RNA immunoprecipitation assay. (D) Relative expression of pnr at 24 and 48 h after dspnr injection. Data are means ± SE (error bars) of four biological replications. U6 or a-tubulin and rps3 were the reference genes used to normalize the expression of miRNA or mRNA. The differences between means were analyzed by Student’s t test. For the significance test: unmarked * indicates not significant. (TIF) [file pgen.1010411.s002.tif]

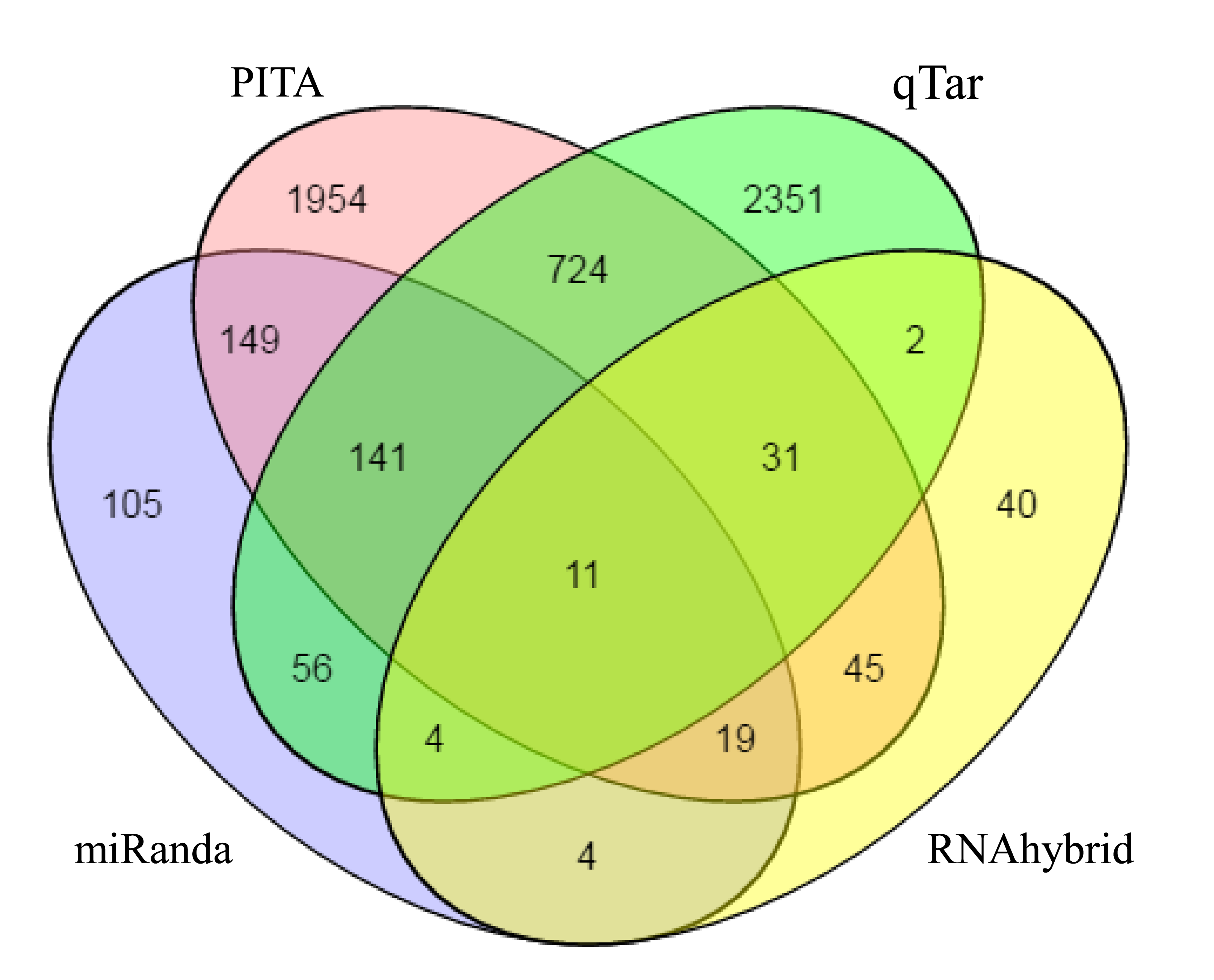

Supplement: S3 Fig — (TIF) [file pgen.1010411.s003.tif]

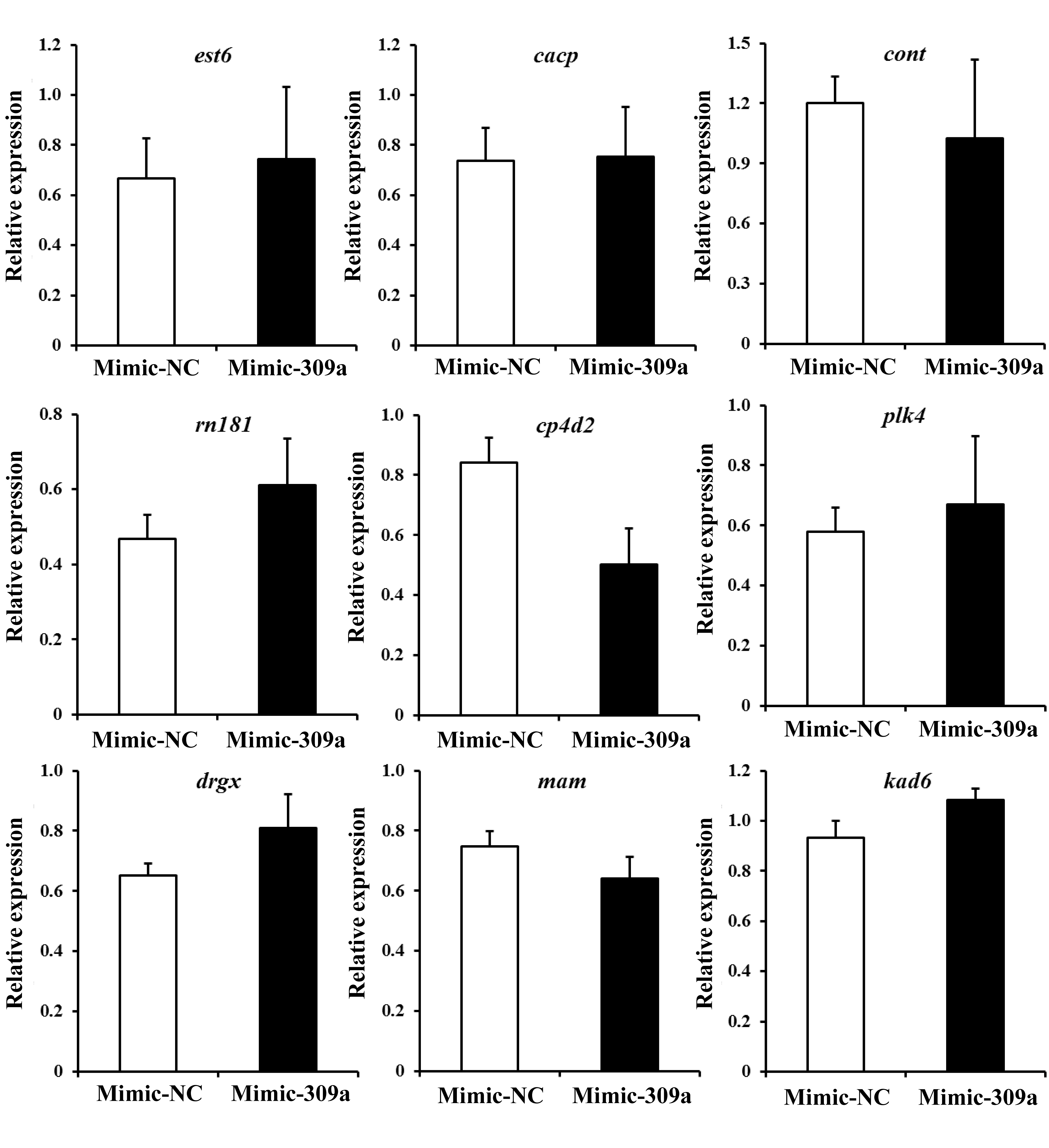

Supplement: S4 Fig — a-tubulin and rps3 were the reference genes used to normalize the expression of mRNA. The differences between means were analyzed by Student’s t test. For the significance test: unmarked * indicates not significant. (TIF) [file pgen.1010411.s004.tif]

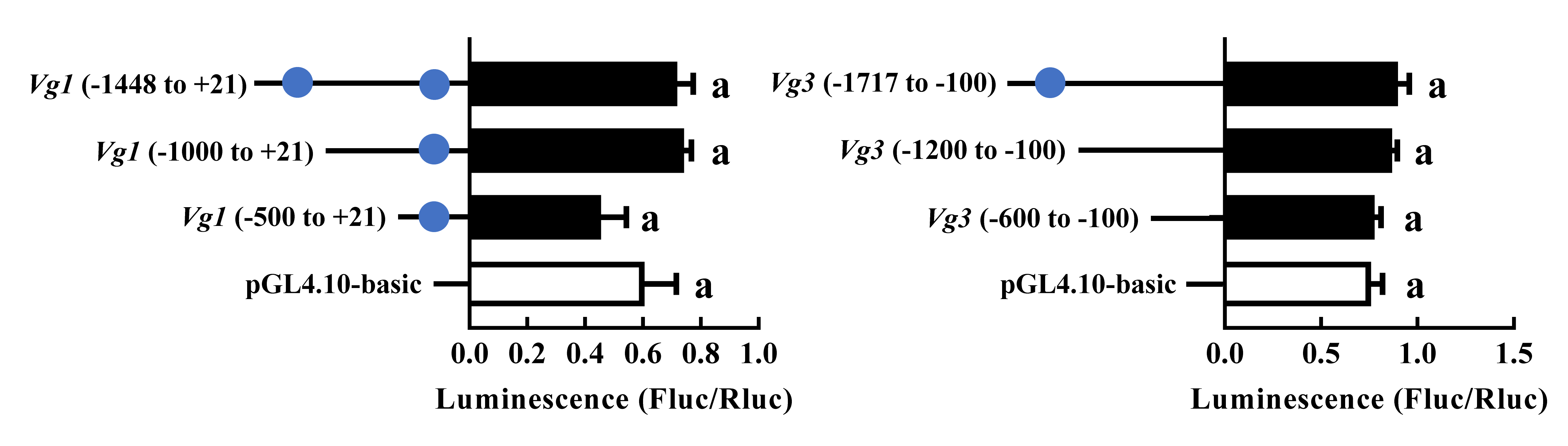

Supplement: S5 Fig — pGL4.10-Vg1−1448 to +21 or pGL4.10-Vg3−1717 to –100 promoter fragments and pGL4.74 were co-transfected into HEK293T cells with the transcription factor pnr in pcDNA3.1-EGFP. pGL4.10-basic vector was used as a control. A blue circle indicates a predicted binding site responding to pnr. Data are means ± SE (error bars) of four biological replications. Same letters above the bars indicate no significant difference among pGL4.10-Vg1 or pGL4.10-Vg3 promoter fragments (Tukey HSD, ANOVA, P < 0.05). (TIF) [file pgen.1010411.s005.tif]

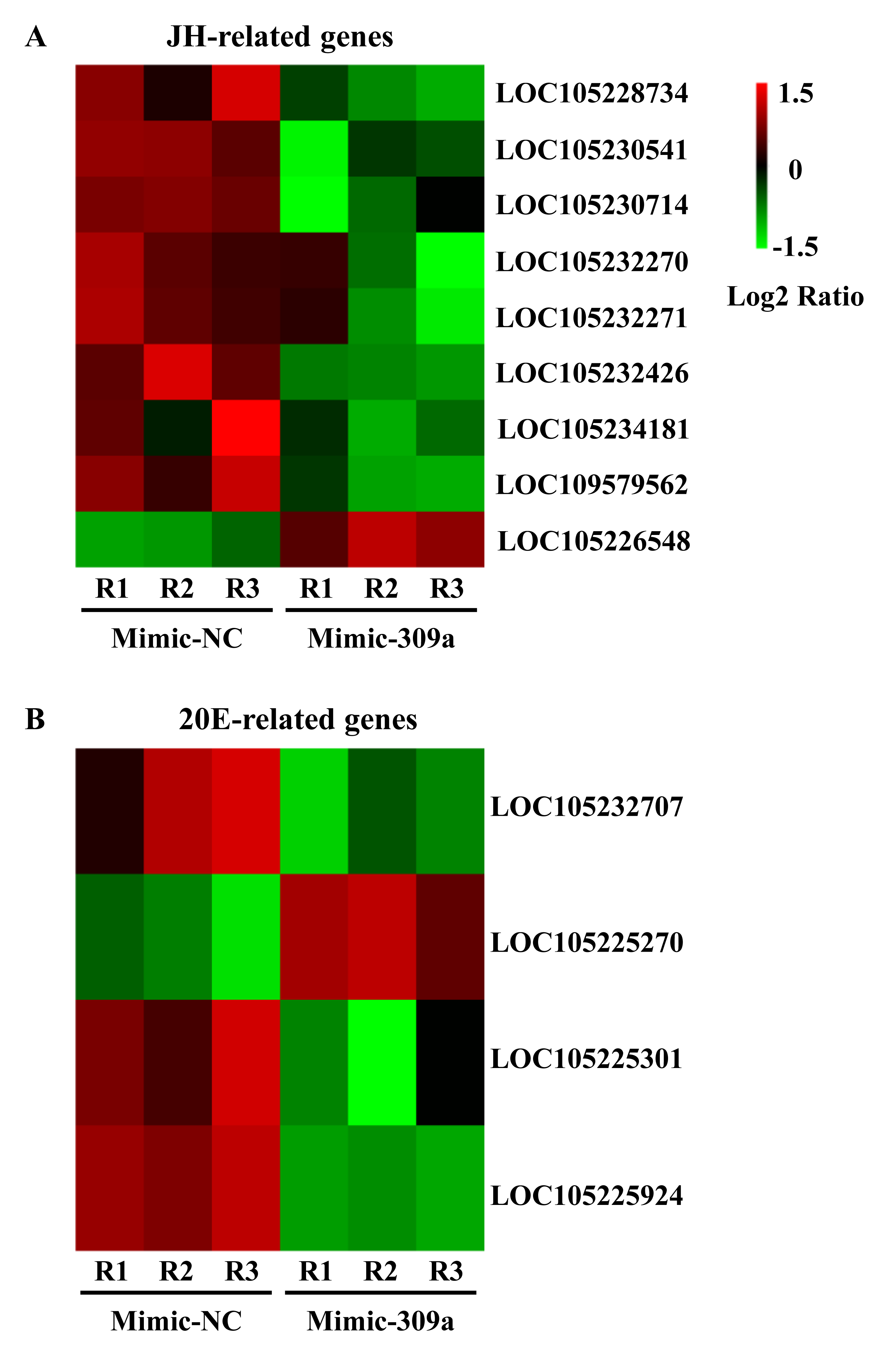

Supplement: S6 Fig — (A) JH-related genes, including significantly down-regulated protein takeout-like (LOC105228734 and LOC105234181), juvenile hormone epoxide hydrolase 2 (LOC105230541), juvenile hormone epoxide hydrolase 2-like (LOC105230714), and uncharacterized protein with juvenile hormone binding protein domain (LOC105232270, LOC105232271, LOC105232426, and LOC109579562) and significantly up-regulated protein takeout-like (LOC105226548). (B) 20E-related genes, including significantly down-regulated cytochrome P450 315a1, mitochondrial (LOC105232707), cytochrome P450 307a1-like (LOC105225301), and ecdysone 20-monooxygenase (LOC105225924) and significantly up-regulated ecdysone-induced protein 74EF (LOC105225270). For the significance test: false discovery rate (FDR, an adjusted P-value) < 0.001 with log2|Fold change| > 2. R1, R2, and R3 indicate three independent biological replications. The color code indicates the fold change of the gene abundance in the form of a logarithm. The fragments per kilobase of exon per million fragments mapped (FPKM) values of genes are normalized in each row. (TIF) [file pgen.1010411.s006.tif]

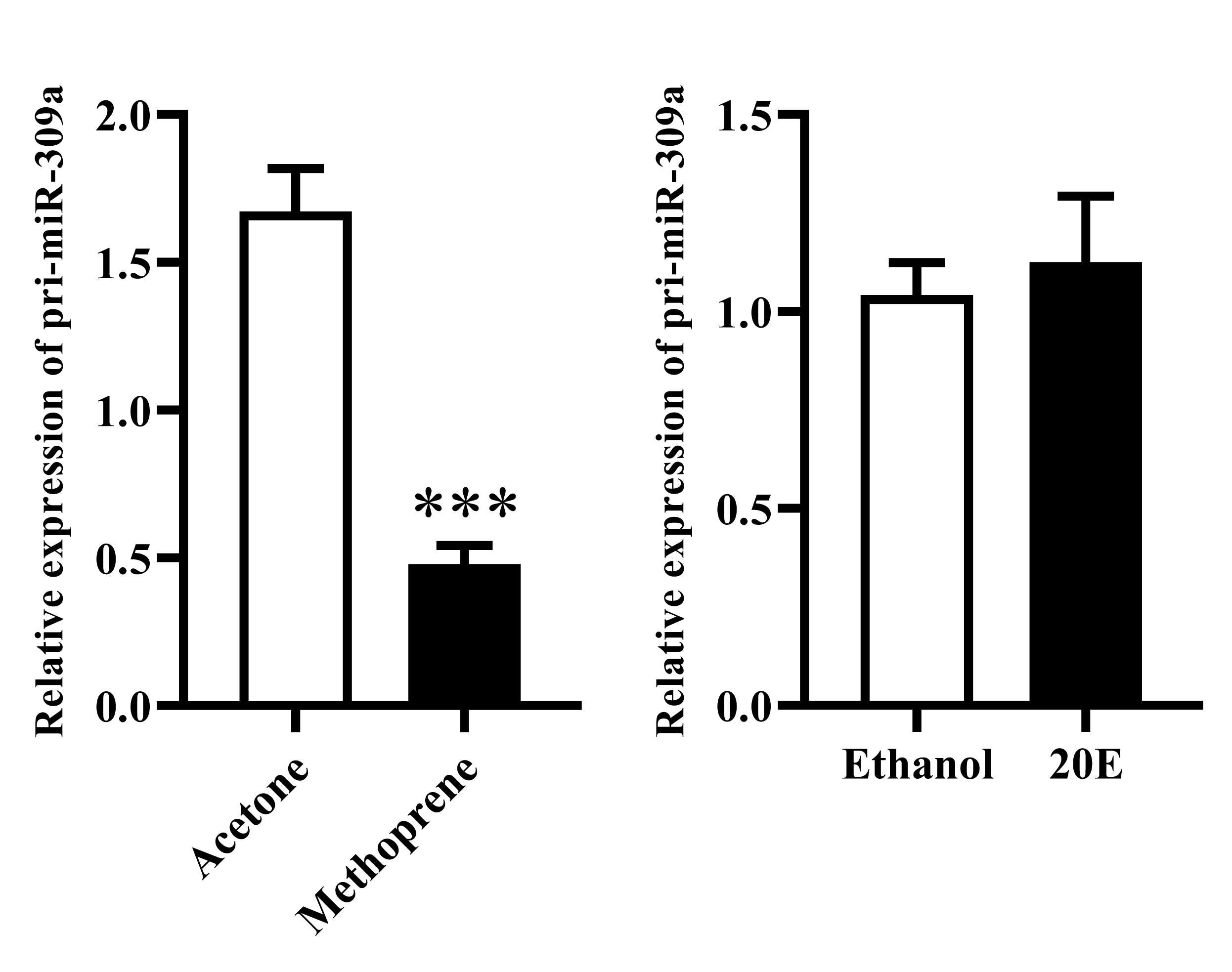

Supplement: S7 Fig — Data are means ± SE (error bars) of four biological replications. a-tubulin and rps3 were the reference genes used to normalize the expression of pri-miR-309a. The differences between means were analyzed by Student’s t test. For the significance test: unmarked * indicates not significant; ***P < 0.001. (TIF) [file pgen.1010411.s007.tif]

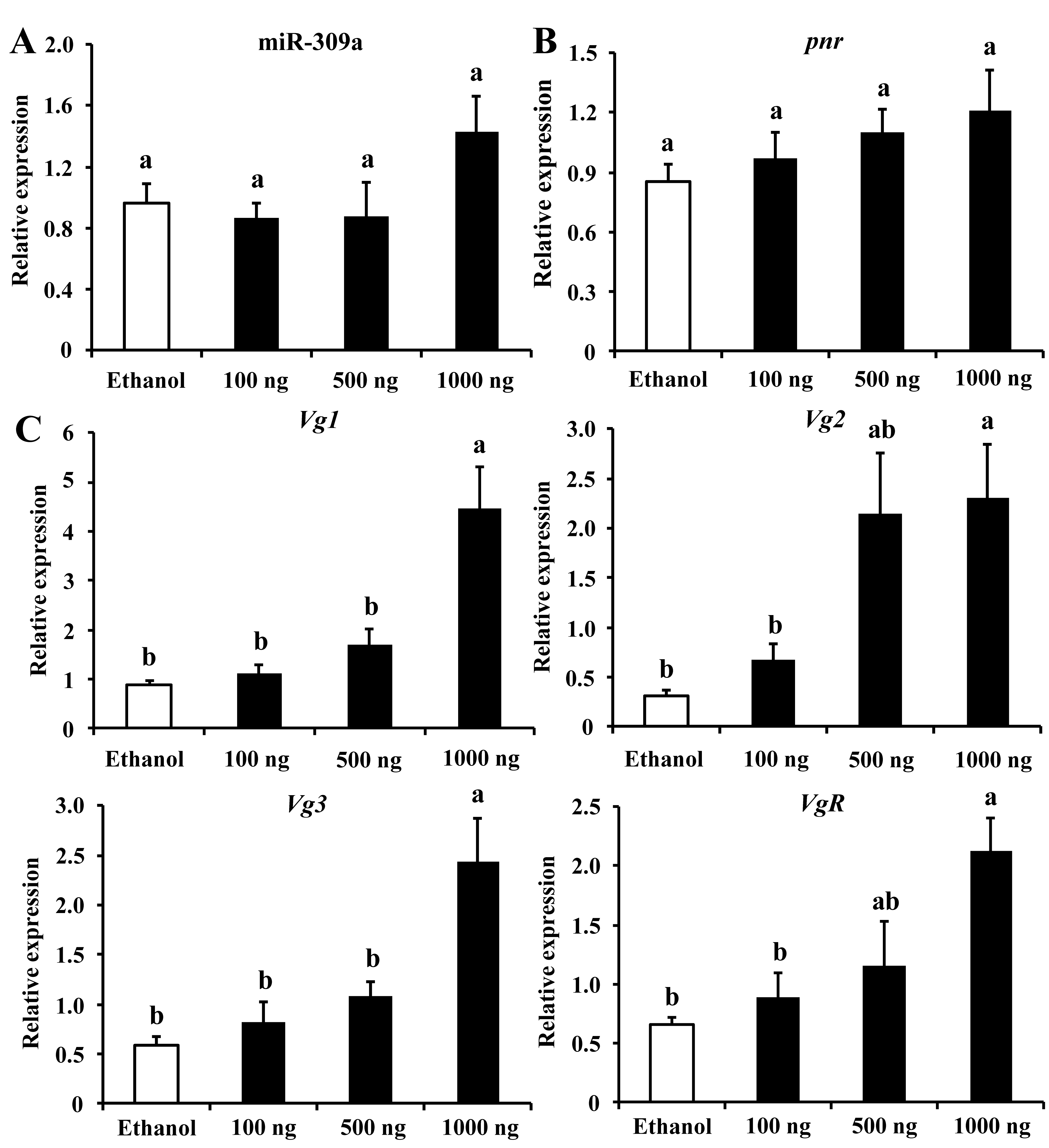

Supplement: S8 Fig — (A) Relative expression of miR-309a. (B) Relative expression of pnr. (C) Relative expression of Vg-related genes. Data are means ± SE (error bars) of four biological replications. U6 or a-tubulin and rps3 were the reference genes used to normalize the expression of miRNA or mRNA. Different letters above the bars indicate significant differences of the miRNA or mRNA at different 20E doses (Tukey’s HSD, ANOVA, P < 0.05). (TIF) [file pgen.1010411.s008.tif]

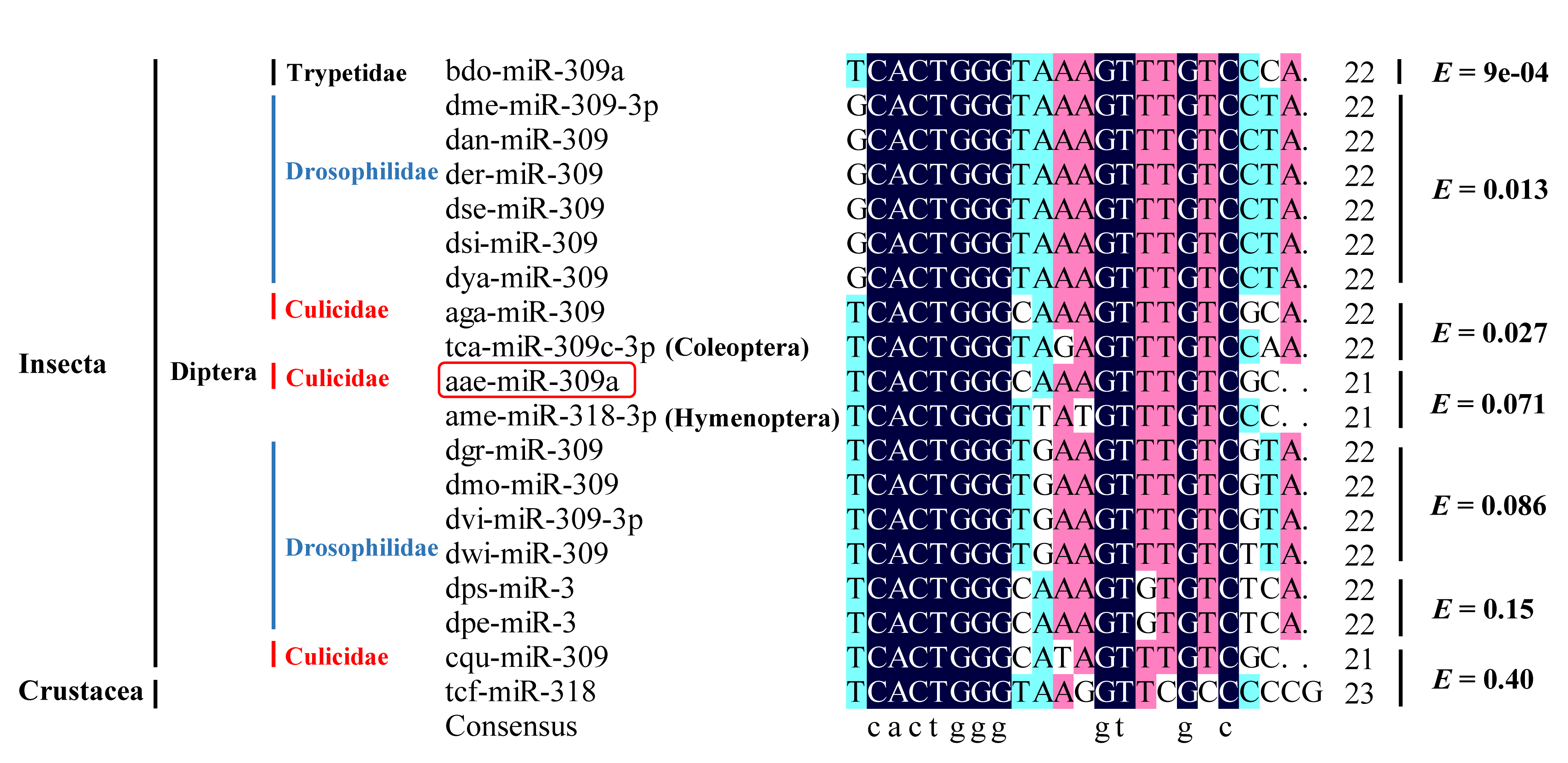

Supplement: S9 Fig — The homology analysis was conducted by blasting in the miRBase database. E indicates E-value. (TIF) [file pgen.1010411.s009.tif]
