# Supplementary material for: miR-309a is a regulator of ovarian development in the oriental fruit fly Bactrocera dorsalis
Source: PLoS Genet. 2022 Sep 16;18(9):e1010411. doi: 10.1371/journal.pgen.1010411 (PMC9518882; doi:10.1371/journal.pgen.1010411)
Supplement: S1 Table — (DOCX) [file pgen.1010411.s010.docx]

**Supplementary Table 1. Primer sequences used in this study.**

| Experiments | | Primer | | | | Nucleotide sequence (5’-3’) | Amplification efficiency (%) |
| --- | --- | --- | --- | --- | --- | --- | --- |
| qPCR | | miR-309a-F | | | | TCACTGGGTAAAGTTTGTCCCA | 98.5 |
|  | | pri-miR-309a-F | | | | AATGGTCACTGCCACCTAAAA | 103.6 |
|  | | pri-miR-309a-R | | | | GATGCAGAAAACAAGCACCA |  |
|  |  | *pnr*-F | | | | AGACCCGCCATTCAACCATT | 99.1 |
|  |  | *pnr*-R | | | | TGGAGATAACGTGTGAGCCG |  |
|  |  | *tret*-F | | | | TTCGGGCGTTCTCTCTCAAC | 100.5 |
|  |  | *tret*-R | | | | CATAGGTCAATACCGGCGCT |  |
|  |  | *est6*-F | | | | AACACTTTTCGGCTACAGCG | 102.7 |
|  |  | *est6*-R | | | | TGAAGGCTCTTTCGGGCAAT |  |
|  |  | *cacp*-F | | | | GGGTAAGGGTGTGGATCGTC | 98.3 |
|  |  | *cacp*-R | | | | CAAGCATAACCATCGTCCGC |  |
|  |  | *cont*-F | | | | TACCACGCAAGAGCACGATT | 99.2 |
|  |  | *cont*-R | | | | AGTCACCACCGTCAAGCAAT |  |
|  |  | *drgx*-F | | | | CTACCGGGCGACCTCTTAAC | 100.1 |
|  |  | *drgx*-R | | | | CAAACCTGTACACGCGCTTC |  |
|  |  | *kad6*-F | | | | CATAACAACCGCTGGAACGC | 98.9 |
|  |  | *kad6*-R | | | | CTCGGGAATGCTGTCGGAAT |  |
|  |  | *mam*-F | | | | CGGCCTTGGCTCAAGTATCA | 101.5 |
|  |  | *mam*-R | | | | TCCCCTCCTCCTCCTCCTAT |  |
|  |  | *rn181*-F | | | | CCGGCGGAAATCGATGAGAA | 98.9 |
|  |  | *rn181*-R | | | | ACGGTTAGACTCGTCTTGGC |  |
|  |  | *cp4d2*-F | | | | CCGATTGCTCTCACGCAAAG | 97.9 |
|  |  | *cp4d2*-R | | | | GCCATACGTCGTTTCAAGCC |  |
|  |  | *plk4*-F | | | | TTCGTCCACCCACTTTAGCC | 97.8 |
|  |  | *plk4*-R | | | | TGACCGTTATGCGTCTGCTT |  |
|  |  | *Vg1-*F | | | | GGAAGAACCCCAAACCCACA | 99.5 |
|  |  | *Vg1-*R | | | | GCACCAACACCTTGTCCAAC |  |
|  |  | *Vg2-*F | | | | CAGGAAGGAGAGCGTTTGATTGG | 98.8 |
|  |  | *Vg2-*R | | | | CTGTTGTCCGTAGTAGCGTTGC |  |
|  |  | *Vg3-*F | | | | CCCAGTCCCAGCGATATTCC | 98.5 |
|  |  | *Vg3-*R | | | | ACTTGTCCGTTGTAGGCCTG |  |
|  |  | *VgR-*F | | | | CTTGCAAGCGTTGGACTACA | 100.3 |
|  |  | *VgR-*R | | | | CCGAAGAGTTGACAATGCAC |  |
| miRNA mimic/antagomir synthesis | | Mimic miR-309a-Sense | | | | UCACUGGGUAAAGUUUGUCCCA | — |
|  |  | Mimic miR-309a-Antisense | | | | UGGGACAAACUUUACCCAGUGA |  |
|  |  | Antagomir miR-309a | | | | UGGGACAAACUUUACCCAGUGA | — |
|  |  | Mimic control-Sense | | | | UUUGUACUACACAAAAGUACUG | — |
|  |  | Mimic control- Antisense | | | | CAGUACUUUUGUGUAGUACAAA |  |
|  |  | Antagomir control | | | | CAGUACUUUUGUGUAGUACAA | — |
| dsRNA synthesis | | ds*pnr-*F | TAATACGACTCACTATAGGGCCAGCACAGCGTCATCTCTA | | | | — |
|  |  | ds*pnr-*R | TAATACGACTCACTATAGGGAGCCGCCAAAGTAGCAGTTA | | | |  |
| Double digestion | | SacI-*pnr*-F | CGAGCTCAGTTTGTCGATAACAGAATGCG | | | | — |
|  |  | XhoI-*pnr*-R | CCGCTCGAGTTTTGTGGAGAGTTGTTGTTG | | | |  |
|  |  | SacI-*tret-*F | CGAGCTCCGGTGAACATCGAGAGATTAG | | | | — |
|  |  | XhoI-*tret*-R | CCGCTCGAGATGAAGCCAAAATCGGTAAAT | | | |  |
| Mutant construction | | Mut-*pnr*-F | AACGCCTCAAACTTCG GGGTCACATCATCATTCCCTGCA | | | | — |
|  |  | Mut-*pnr*-R | TGCAGGGAATGATGATGTGACCCCGAAGTTTGAGGCGTT | | | |  |
| Promoter analysis | | *Vg1*-promoter-F | | ATGTTGACATATAACGAGTGATC | | | — |
|  |  | *Vg1*-promoter-R | | GAAAATCTTTAGAGGATTCAT | | |  |
|  | | *Vg2*-promoter-F | | AATGTTGATATTTGAATAGGAAAAT | | | — |
|  | | *Vg2*-promoter-R | | CGCTGACGTGCTGTATGGTT | | |  |
|  | | *Vg3*-promoter-F | | GCAGCACATGGGGAAAAGAC | | | — |
|  | | *Vg3*-promoter-R | | GCGTAGCACAACTCAGAATGC | | |  |
|  | | *VgR*-promoter-F | | ACGCCTACTTTTAACACTAATATAAT | | | — |
|  | | *VgR*-promoter-R | | TTGAAAACTTACATTGATTGCC | | |  |
|  | pcDNA3.1-EGFP-*pnr*-F | | | | ACGAGCTGTACAAGTAAAGCGGCCGCATGGGCATACTAATGATTGCAA | | — |
|  | pcDNA3.1-EGFP-*pnr*-R | | | | GCGGGTTTAAACGGGCCCTCTAGATTACGATGTGGCCATTAACTTG | |  |

The underlined part of primer sequence indicates T7 promoter sequence. F: forward primer, R: reverse primer.
